# Assessing thresholds of resistance prevalence at which empiric treatment of gonorrhea should change among men who have sex with men in the US: A cost-effectiveness analysis

**DOI:** 10.1371/journal.pmed.1004424

**Published:** 2024-07-08

**Authors:** Xuecheng Yin, Yunfei Li, Minttu M. Rönn, Song Li, Yue Yuan, Thomas L. Gift, Katherine Hsu, Joshua A. Salomon, Yonatan H. Grad, Reza Yaesoubi

**Affiliations:** 1 Department of Management Science and Information Systems, Spears School of Business, Oklahoma State University, Tulsa, Oklahoma, United States of America; 2 Department of Health Policy and Management, Yale School of Public Health, New Haven, Connecticut, United States of America; 3 Yale Public Health Modeling Unit, Yale School of Public Health, New Haven, Connecticut, United States of America; 4 Department of Global Health and Population, Harvard T.H. Chan School of Public Health, Boston, Massachusetts, United States of America; 5 College of Computer Science and Technology/School of Cyber Science and Technology, Zhejiang University, Hangzhou, China; 6 Altfest Personal Wealth Management, New York, New York, United States of America; 7 Division of STD Prevention, Centers for Disease Control and Prevention, Atlanta, Georgia, United States of America; 8 Massachusetts Department of Public Health, Boston, Massachusetts, United States of America; 9 Department of Health Policy, Stanford University School of Medicine, Stanford, California, United States of America; 10 Department of Immunology and Infectious Diseases, Harvard T. H. Chan School of Public Health, Boston, Massachusetts, United States of America

## Abstract

**Background:**

Since common diagnostic tests for gonorrhea do not provide information about susceptibility to antibiotics, treatment of gonorrhea remains empiric. Antibiotics used for empiric therapy are usually changed once resistance prevalence exceeds a certain threshold (e.g., 5%). A low switch threshold is intended to increase the probability that an infection is successfully treated with the first-line antibiotic, but it could also increase the pace at which recommendations are switched to newer antibiotics. Little is known about the impact of changing the switch threshold on the incidence of gonorrhea, the rate of treatment failure, and the overall cost and quality-adjusted life-years (QALYs) associated with gonorrhea.

**Methods and findings:**

We developed a transmission model of gonococcal infection with multiple resistant strains to project gonorrhea-associated costs and loss in QALYs under different switch thresholds among men who have sex with men (MSM) in the United States. We accounted for the costs and disutilities associated with symptoms, diagnosis, treatment, and sequelae, and combined costs and QALYs in a measure of net health benefit (NHB). Our results suggest that under a scenario where 3 antibiotics are available over the next 50 years (2 suitable for the first-line therapy of gonorrhea and 1 suitable only for the retreatment of resistant infections), changing the switch threshold between 1% and 10% does not meaningfully impact the annual number of gonorrhea cases, total costs, or total QALY losses associated with gonorrhea. However, if a new antibiotic is to become available in the future, choosing a lower switch threshold could improve the population NHB. If in addition, drug-susceptibility testing (DST) is available to inform retreatment regimens after unsuccessful first-line therapy, setting the switch threshold at 1% to 2% is expected to maximize the population NHB. A limitation of our study is that our analysis only focuses on the MSM population and does not consider the influence of interventions such as vaccine and common use of rapid drugs susceptibility tests to inform first-line therapy.

**Conclusions:**

Changing the switch threshold for first-line antibiotics may not substantially change the health and financial outcomes associated with gonorrhea. However, the switch threshold could be reduced when newer antibiotics are expected to become available soon or when in addition to future novel antibiotics, DST is also available to inform retreatment regimens.

## Abstract 2

Using a mathematical model of gonococcal infection, Xuecheng Yin and co-authors project the burden of gonorrhea in a population of men who have sex with men in the US and, the gonorrhea-associated costs and loss in quality adjusted life-years under various antibiotic switch thresholds and scenarios for antibiotic availability.

## Author summary

**Why was this study done?:**

- Antibiotics used for the empiric therapy of gonorrhea are usually changed once the prevalence of resistance to the antibiotic exceeds a certain threshold, currently set at 5%.
- A low switch threshold is often selected to ensure that the first-line antibiotic remains effective for most patients with gonorrhea.
- However, little is known about the impact of changing the switch threshold on the incidence of gonorrhea, the rate of treatment failure, and the overall cost and quality-adjusted life-years (QALYs) associated with gonorrhea.

**What did the researchers do and find?:**

- We developed a mathematical model of gonococcal infection among a population of men who have sex with men (MSM) in the United States to project the burden of gonorrhea and the overall associated cost and QALYs under various switch thresholds and scenarios for the future availability of antibiotics and drug-susceptibility testing (DST).
- We found that changing the switch threshold between 1% and 10% does not meaningfully impact the annual number of gonorrhea cases, and total cost and total QALY loss associated with gonorrhea.
- However, if a new antibiotic is expected to become available in the future choosing a lower threshold could improve the population net health benefit (NHB).

**What do these findings mean?:**

- Changing the switch threshold may not substantially impact the health and financial outcomes associated with gonorrhea. However, the switch threshold could be reduced when newer antibiotics are expected to become available soon or when DSTs is available to inform retreatment regiments.
- Our study was limited to MSM in the US and future studies should evaluate the generalizability of our findings to other populations.

## Introduction

Gonorrhea, caused by the pathogen *Neisseria gonorrhoeae*, is one of the most common notifiable diseases with 677,769 reported cases in 2020 in the United States [1] and an estimated 87 million incident cases worldwide in 2016 [2,3]. *N*. *gonorrhoeae* has developed resistance to each of the first-line antibiotics recommended to treat it. Due to the high prevalence of gonorrhea among certain groups (e.g., men who have sex with men (MSM)) and increasing antibiotic resistance, the US Centers for Disease Control and Prevention (CDC) named antimicrobial-resistant (AMR) gonorrhea one of the 3 most urgent antimicrobial resistance threats in the United States [4].

Gonorrhea is most commonly diagnosed by a nucleic acid amplification test, which does not routinely provide results on antibiotic susceptibility. Therefore, the treatment of gonorrhea remains empiric and based on standardized treatment guidelines [5]. These guidelines are determined based on the estimated prevalence of resistance reported by national surveillance systems such as the Gonococcal Isolate Surveillance Project (GISP) in the US [6]. Currently, the guidelines recommend ceftriaxone for the first-line treatment of uncomplicated gonorrhea [5]. The percentage of GISP isolates that exhibited resistance to ceftriaxone has fluctuated around 0.2% between 2016 and 2020 [7]. To ensure the effectiveness of empiric treatment, guidelines recommend using antibiotics with a prevalence of resistance below 5% and to switch to a new antibiotic for first-line therapy when the prevalence of resistance exceeds this threshold [8,9]. However, the evidence to support this threshold is not clear, and the impact of changing this switch threshold on the gonorrhea-related outcomes (e.g., number of gonorrhea cases or treatment failure rate) has not been studied before.

Increasing the threshold delays switching to a new antibiotic, which decreases the probability that standardized first-line therapy is effective for a gonococcal infection. This could result in many individuals with AMR gonorrhea receiving ineffective therapy, experiencing a lower quality of life while symptomatic, and contributing to the spread of AMR gonorrhea. Decreasing the threshold could improve the effectiveness of empiric therapy, but also would lead to earlier and more extensive use of second-line regimens, which could shorten their life span and lead to the emergence of additional resistance [9].

To evaluate the health and cost consequences of different switch thresholds, we developed a simulation model of gonococcal transmission among MSM in the US. We used this model to project the cost and quality-adjusted life-years (QALYs) loss due to gonorrhea among MSM over 50 years under different switch thresholds. We conducted cost-effectiveness analyses to identify the optimal switch threshold under different scenarios representing the availability and cost of novel antibiotics and drug-susceptibility testing (DST). We conducted our cost-effectiveness analyses from a healthcare sector perspective, which accounts for medical costs incurred by healthcare payers or patients including the costs of diagnosis, treatment, and sequela associated with gonococcal infection.

## Methods

### Simulation model

We considered a scenario in which 2 antibiotics that are suitable for the first-line treatment of gonorrhea are available (Drug A and Drug B). Drug A represents ceftriaxone, which is currently recommended by CDC for empiric treatment of gonorrhea [10], and Drug B represents a future antibiotic, which is assumed to be comparable with ceftriaxone in terms of cost, side effects, and the fitness cost, defined as the relative infectiousness compared to the drug-susceptible strain. We assumed that initially Drug A is used for empiric treatment of gonorrhea and that Drug B will replace Drug A as first-line therapy when the prevalence of resistance to Drug A exceeds a certain threshold (e.g., 5%).

**Fig 1:**
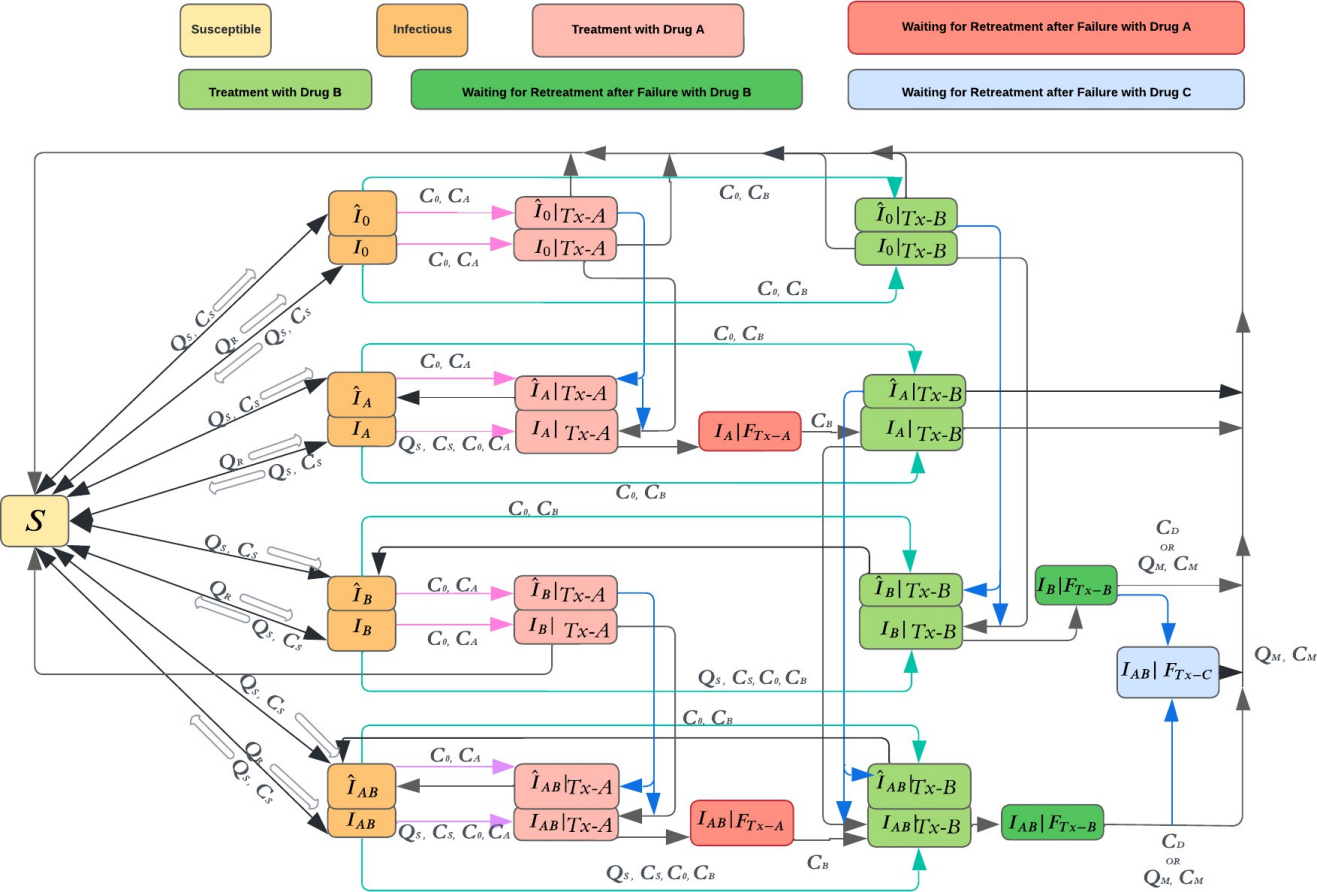
A stochastic compartmental model of gonorrhea transmission among MSM in the United States. The asymptomatic compartments are denoted by ^ symbol. The transitions from infectious states to treatment states are represented by light red arrows when Drug A is used as first-line therapy and by light green arrows when Drug B is used as first-line therapy. Drug C represents a future antibiotic. When it becomes available, if Drugs A or B is still used for first-line therapy, Drug C will be reserved for second-line therapy; however, if Drugs A and B are removed due to their resistance passing the specified switch threshold, Drug C will be used as first-line therapy (see Modeling the introduction of a new antibiotic section in S1 Appendix). The development of resistance during treatment is represented by blue arrows. The variables above arrows represent the cost (C) and health (Q) outcomes due to transition between health states (see Tables 1 and 2). MSM, men who have sex with men.

We developed a compartmental model to simulate transmission of gonococcal infection among the MSM population of age 14 years and above in the US (Fig 1). In 2018, the estimated number of reported gonorrhea cases was 6,508 [5,207, 7,809] per 100,000 MSM population, and the emergence of resistance among this population is of particular concern [11,12]. We divided the population into 3 groups based on levels of sexual activity (low, intermediate, and high) [13,14], represented by rates of partner change. We assumed that individuals remained in their assigned risk group for the duration of their time in the model. We allowed for mixing within and between sexual-behavior groups based on the approach proposed by [15] and used in other modeling studies related to sexually transmitted infections [13,14]. The details are discussed in Additional model details section in S1 Appendix.

In our model, susceptible individuals (*S* in Fig 1) can become infected with a probability that varies based on the prevalence of gonococcal infections. An infected individual may be symptomatic or asymptomatic (*I* versus $\hat{I}$ in Fig 1). There are 4 possible infection resistance profiles among gonococcal strains: susceptible to both Drugs A and B ($I_{0},{\hat{I}}_{0}$), susceptible to Drug A but resistant to Drug B ($I_{B},{\hat{I}}_{B}$), susceptible to Drug B but resistant to Drug A ($I_{A},{\hat{I}}_{A}$), and resistant to both Drugs A and B ($I_{AB},{\hat{I}}_{AB}$). Infected individuals may spontaneously recover from infection and return to the susceptible compartment, seek treatment for their symptoms, or get detected through screening for asymptomatic infection and receive treatment.

Those who seek treatment or are identified through screening receive treatment with the first-line antibiotic. The majority of individuals with infection susceptible to the first-line antibiotics are successfully treated and return to the susceptible compartment. A portion of these individuals, however, develop resistance to the first-line antibiotic during the treatment. An individual with asymptomatic infection might become symptomatic if resistance is developed during treatment. If first-line therapy fails due to using antibiotics that do not match the resistance profile or due to the development of resistance during treatment, symptomatic individuals seek retreatment with some delay and move to compartments ${I_{A}|F}_{\mathrm{Tx}‐A},{I_{B}|F}_{\mathrm{Tx}‐B},{I_{AB}|F}_{\mathrm{Tx}‐A}$, or *I*_*AB*_|*F*_*Tx*-*B*_ depending on their prior resistance profile. Asymptomatic individuals, however, do not seek retreatment and will remain infectious and move back to compartments ${\hat{I}}_{A}$ or ${\hat{I}}_{AB}$.

When Drug A is used as the first-line antibiotic, infected individuals with identified unsuccessful treatment with Drug A will receive Drug B. However, when Drug B is used for first-line therapy, individuals with identified unsuccessful first-line therapy will be treated with Drug M. Drug M represents ertapenem, which is used for the treatment of serious infections and is not suitable for empiric treatment due to cost and side effects. We assumed that those who treated with Drug M will recover and move to the susceptible compartment.

To model the impact of new first-line antibiotics (in addition to Drugs A and B), we used the following approach. If a new antibiotic (say, Drug C) becomes available when the resistance of prevalence for current first-line antibiotic is still below the selected switch threshold, Drug C will be reserved for second-line therapy (i.e., it will be used in place of Drug M to treat infections with resistance to B or both Drugs A and B). A small portion of infections with resistance to Drugs A and B that are treated with Drug C may also develop resistance to Drug C and move to the compartment *I*_*AB*_|*F*_*Tx*-*C*_. We assumed that individuals in this compartment do not contribute to the overall force of infection due to reduced sexual activity and will be successfully treated after receiving Drug M. When the prevalence of resistance to Drug B passes the selected switch threshold, Drug C will replace Drug B in first-line therapy. Since our model includes only 2 first-line antibiotics (Drugs A and B), the introduction of a new Drug C requires redefining Drugs A and B such that Drug A now represents the previous first-line antibiotic (i.e., Drug B) and Drug B now represents the new Drug C (which is used as second-line therapy). This is implemented by moving members of model compartments according to certain rules, which are described in Modeling the introduction of a new antibiotic section in S1 Appendix.

### Model calibration

We used a Bayesian approach to calibrate our model against gonorrhea prevalence, annual case rate, and the proportion of asymptomatic infected individuals [16]. The estimates for these calibration targets were extracted from existing studies [17–19]. This calibration approach aimed to estimate the probability distributions of unknown parameters such that trajectories that are simulated using random draws from these distributions fit the available epidemiological data. The prior distributions for model parameters were obtained from existing estimates. For parameters for which such estimates were not available, we used distributions based on biologically plausible bounds as priors. Additional details on the calibration procedure as well as the distributions and mean values for parameters are provided in Model calibration section in S1 Appendix.

### Evaluating the cost-effectiveness of different switch thresholds

We conducted cost-effectiveness analysis of different switch thresholds using a health sector perspective. Transitions between health states represented in Fig 1 could result in costs (denoted by *C*) and loss in QALYs (denoted by *Q*). We assumed that costs may be incurred due to diagnosis, drug-susceptibility testing, treatment, and sequelae (Table 1) and that those with symptomatic infection, receiving treatment, or developing sequelae would experience lower quality of life than those with asymptomatic infection that did not lead to sequelae (Table 2). We calculated the QALY loss due to transition into a health state as *UJ*, where *U* is the disutility associated with the new state and *J* is the expected stay duration in the new state (Table 2). We assumed that a small portion of individuals who receive an ineffective treatment or recover without treatment may develop sequelae. We considered epididymitis and disseminated gonococcal infection (DGI) as only sequelae with probabilities listed in Table 2. If a transition could lead to sequelae, the expected QALY loss is denoted by *Q*_*S*_ and is calculated according to trees shown in Fig C in S1 Appendix.

**Table 1:** Cost (in 2022 US$) parameters in our model (Fig 1).

Parameter	Description	Mean estimate	Uncertainty interval	Reference
*C*_0_	Cost of diagnosis	$68	$35 to $100	[34]
*C*_*D*_	Cost of DST	$150	$100 to $200	Assumption
*C*_*A*_, *C*_*B*_	Cost of treatment with Drug A or Drug B, which is assumed to be the same as the cost of treatment with ceftriaxone	$65 or $116		Sum of cost items below*
	Ceftriaxone	$24	$12 to $36	[34]
	Short clinic visit	$41	$21 to $61	[34]
	Treatment of urethritis	$92	$48 to $136	[34]
*C*_*M*_	Cost of treatment with Drug M, which is assumed to be the same as the cost of treatment with Ertapenem	$578 or $629		Sum of cost items below*
	Ertapenem	$537	$291 to $782	[35]
	Short clinic visit	$41	$21 to $61	[34]
	Treatment of urethritis	$92	$48 to $136	[34]
*C*_*S*_	Expected cost of sequelae	$53		Calculated according to the probability tree in Fig C in S1 Appendix.
	Cost of epididymitis (*C*_*E*_)	$522	$268 to $775	[34]
	Cost of DGI (*C*_*D*_)	$2,916	$1,160 to $4,672	[34]
	Cost of epididymitis and DGI (*C*_*ED*_)	$3,438		Sum of 2 cost items above
	Probability of epididymitis per untreated infection or after treatment failure (*P*_*E*_)	0.042	0.0012 to 0.14	[34]
	Probability of DGI per untreated infection or after treatment failure (*P*_*D*_)	0.01	0.0075 to 0.013	[34]
	Probability of epididymitis and DGI per untreated infection or after treatment failure (*P*_*ED*_)	0.00042	0.00001 to 0.00182	[34]

* We assumed that the cost of treatment depends on whether the infection is symptomatic or asymptomatic. All male individuals with symptomatic gonococcal infection were assumed to have urethritis for the duration of the infection [36]. Therefore, we calculated the cost of treatment for symptomatic infection as the sum of antibiotic cost and the cost of urethritis treatment—consistent with previous studies [34], we assumed that the cost of urethritis treatment was already included in the cost of short clinical visit. The treatment cost of individuals with asymptomatic gonococcal infection was assumed to include the cost of antibiotic and the cost for a short clinic visit.

DGI, disseminated gonococcal infection; DST, drug-susceptibility testing.

**Table 2:** Model parameters determining the loss in QALYs.

Parameter	Description	Mean estimate	Uncertainty interval	Reference
*Q*_*R*_	QALYs lost for symptomatic infection	0.00304		Product of items below divided by 365
	Disutility of symptomatic urethral infection (*U*_*R*_)	0.16	0.08 to 0.24	[34]
	Duration (days) of symptomatic urethral infection (*J*_*R*_)	6.9	3.7 to 10.2	[34]
*Q*_*M*_	QALYs lost for symptomatic infection receiving treatment with Ertapenem	0.00863		Product of items below divided by 365
	Disutility while receiving treatment (*U*_*M*_)	0.3	0.2 to 0.4	[37] (assumption)
	Duration (days) of treatment (*J*_*M*_)	10.5	7 to 14	[38]
*Q*_*S*_	Expected QALYs lost of sequelae	0.00053		Calculated according to the probability tree shown in Fig C in S1 Appendix.
	Disutility of epididymitis (*U*_*E*_)	0.54	0.28 to 0.8	[34]
	Disutility of DGI (*U*_*D*_)	0.37	0.19 to 0.55	[34]
	Disutility of epididymitis and DGI (*U*_*ED*_)	0.7102		Calculated as 1−(1−*U*_*E*_)(1−*U*_*D*_)
	Duration (days) of epididymitis (*J*_*E*_)	6.9	3.6 to 10.2	[34]
	Duration (days) of DGI (*J*_*D*_)	8.8	4.7 to 13.1	[34]

DGI, disseminated gonorrhea infection; QALY, quality-adjusted life-years.

We used gamma and beta distributions to account for the uncertainties in cost and utility parameters (Tables 1 and 2). To characterize these distributions, we used an optimization process to match the mean and 95th percentile intervals from the distribution with the mean and uncertainty interval [*a*, *b*] reported in Tables 1 and 2.

We then simulated trajectories with random draws of the cost, disutility, and duration parameters. We discounted the overall costs and QALYs to year 2022 at a rate of 3% per year. All costs estimates are presented in 2022 US dollars using the annual inflation rate of 2.2% based on the Personal Health Care (PHC) price indices [20].

We defined an optimal switch threshold under each scenario of antibiotic availability as the one that minimizes the loss in the population net health benefit (NHB) compared with the status quo scenario of using the 5% switch threshold [21]. For each switch threshold, we calculated the discounted loss in population NHB: (discounted loss in QALYs associated with gonorrhea) + (discounted cost associated with gonorrhea)/ω, where ω is the decision-maker’s willingness-to-pay (WTP) to gain 1 QALY [21].

We used the model to project the costs and losses in QALYs over a 50-year simulation period. The simulation window of 50 years was selected to ensure enough time for the resistance against Drug A and Drug B to develop. We summarized the results using the mean and 95% uncertainty interval (i.e., the interval between 2.5th and 97.5th percentiles of realizations) across 1,000 simulated trajectories. We conducted our CEA following the CHEERS guideline. The CHEERS checklist is included in S1 Checklist.

### Scenario and sensitivity analyses

For our “Base” scenario, we assumed that only Drugs A and B are available for the first-line therapy of gonorrhea over the next 50 years. In this scenario, those with unsuccessful first-line treatment will be empirically treated with the second-line drug; that is, Drug B when Drug A is used for first-line therapy, and Drug M or Drug C (if available) when Drug B is used for first-line therapy. To investigate how future uncertainties related to antibiotic availability and access to DST would impact our conclusions, we considered 3 additional scenarios. The “Base + DST for retreatment” scenario is the same as Base but assumed that the results of DST are used to select the antibiotic for the retreatment of individuals with unsuccessful first-line therapy. We considered 2 other scenarios which are the same as “Base” and “Base + DST for retreatment” except that a new antibiotic will become available at year 30. We referred to these scenarios as “Base + new antibiotic at year 30” and “Base + DST for retreatment + new antibiotic at year 30.”

We also investigated how changing the transmissibility of resistant strains, the probability that additional resistance developed under treatment, and the costs of antibiotics would impact our conclusions (see Sensitivity analysis section in S1 Appendix).

## Results

**Fig 2:**
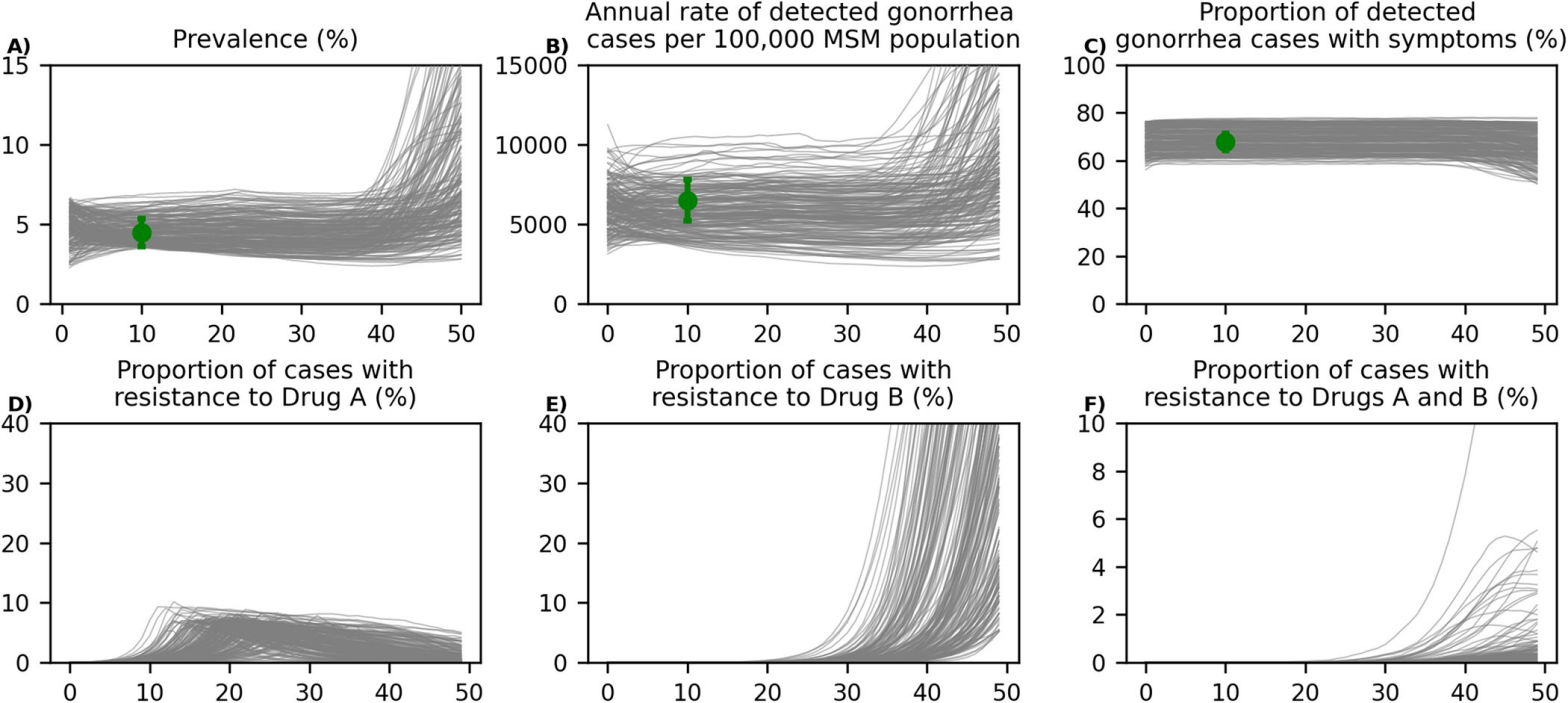
Displaying random 200 simulated trajectories from the calibrated model. The green dots in panels A–C represent the estimated prevalence of gonorrhea (4.5% [3.6%, 5.4%] among MSM) [17], the estimated number of reported gonorrhea cases per 100,000 population in 2018 (6,508 [5,207, 7,809]) [33], and the proportion of gonorrhea cases that were symptomatic (67.9% [64.4%, 71.4%]) [19] among MSM. In these simulated trajectories, we assume that only Drug A and Drug B are available for first-line therapy over the next 50 years. Drug A is initially used for first-line therapy and when the prevalence of resistance to Drug A passes 5%, Drug B will replace Drug A in first-line therapy. See Fig A in S1 Appendix or the prevalence of infection and the spread of resistance to Drug A and Drug B in different sexual activity groups for these trajectories. MSM, men who have sex with men.

We fitted our model to estimates for gonorrhea prevalence, the rate of annual reported gonorrhea cases, and the proportion of symptomatic gonorrhea cases (Fig 2A–2C). Resistance to Drug A increases until the 5% threshold is reached, which results in switching first-line therapy to use Drug B for empiric treatment (Fig 2D). As more gonococcal infections are treated with Drug B, prevalence of resistance to this drug increases. In simulated trajectories displayed in Fig 2, we assumed that no new antibiotic will become available after Drug B; therefore, the prevalence of resistance to Drug B will continue to increase (Fig 2E). We also noted that the speed at which resistance to Drug A or Drug B spreads differ substantially in trajectories displayed in Fig 2. This is consistent with historical data related to *N*. *gonorrhoeae* resistance. For example, it took only a few years for the resistance to sulfonamides to emerge and spread, while the full resistance to penicillin arrived with the introduction of the beta lactamase, 30 years after penicillin’s introduction [22].

**Fig 3:**
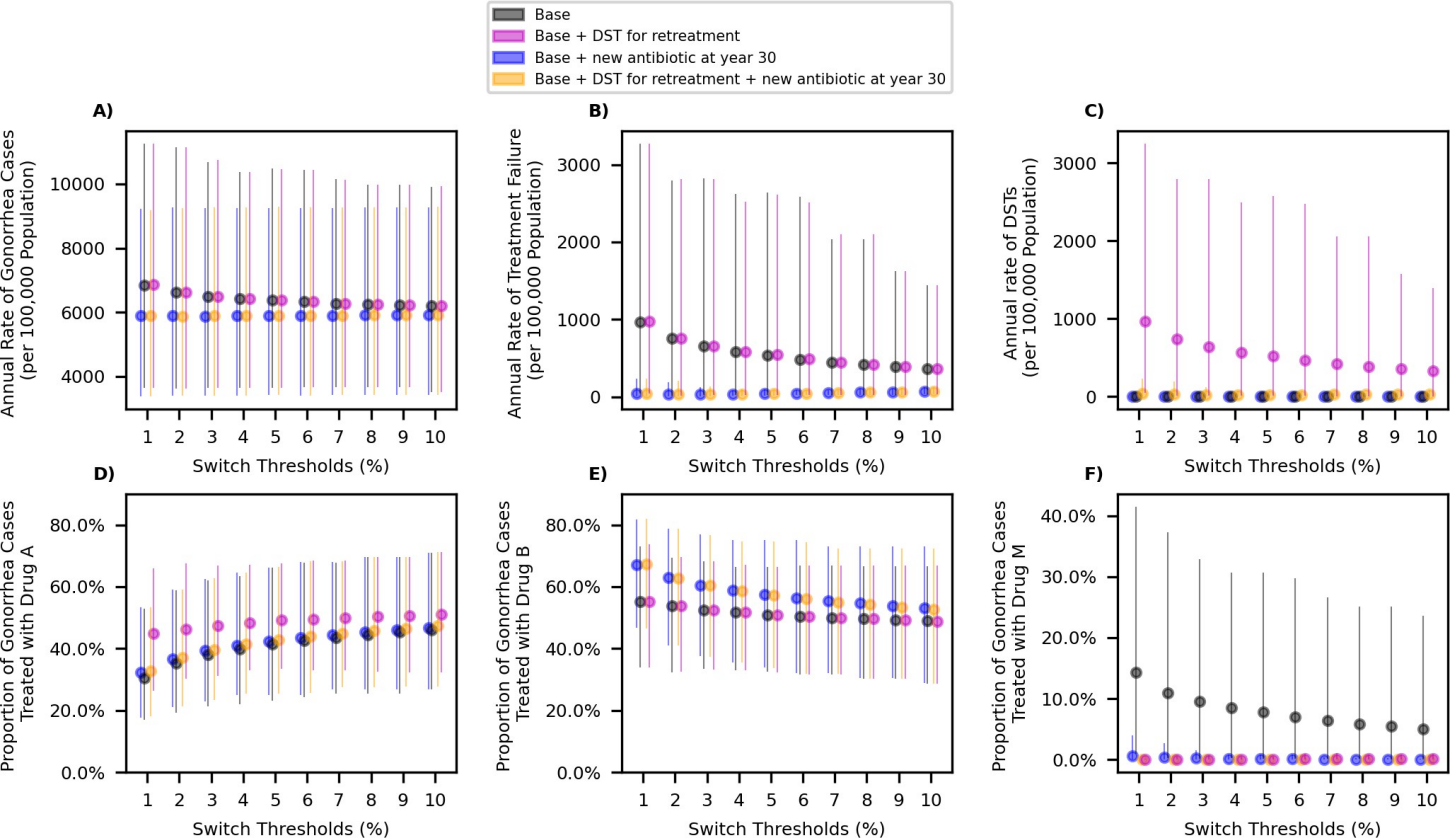
**The impact of changing the switch threshold on the average annual rate of gonorrhea cases (panel A), average annual rate of treatment failure (panel B), the average annual rate of DST (panel C), and the proportion of cases treated with Drug A, Drug B, and Drug M (panels D–F).** Dots represent the mean value and bars represent the 95% uncertainty intervals. Drugs A and B are considered suitable for first-line therapy. Initially, Drug A is used for empiric treatment of gonorrhea and Drug B will replace Drug A when the prevalence of resistance to Drug A exceeds a certain threshold. Drug M is not suitable for first-line therapy and is used only when treatment with Drugs A and B is unsuccessful or when both Drugs A and B are removed from first-line therapy. DST, drug-susceptibility testing.

Changing the switch threshold between 1% and 10% does not substantially change the annual rate of gonorrhea cases, the annual rate of treatment failure, or the annual rate of DSTs performed under scenarios considered here (Fig 3A–3C). Increasing the threshold, however, increases the number of individuals treated with Drug A and decreases the number of individuals treated with Drug B (Fig 3D and 3E); this is because increasing the threshold delays switching from Drug A to Drug B for first-line therapy. The availability of DSTs to inform the retreatment antibiotic reduces the consumption of Drug M when a new antibiotic is not expected to become available (Fig 3F) but does not impact other outcomes markedly. The introduction of a new antibiotic at year 30 is expected to reduce rates of gonorrhea cases and treatment failure as well as reducing demands for DSTs and Drug M (panels A–C and F in Fig 3, comparing black and purple dots to blue and orange dots). Similar behavior is observed for scenarios with higher probability that additional resistance emerges under treatment (Fig F in S1 Appendix) and lower transmissibility of resistant strains (Fig I in S1 Appendix).

**Fig 4:**
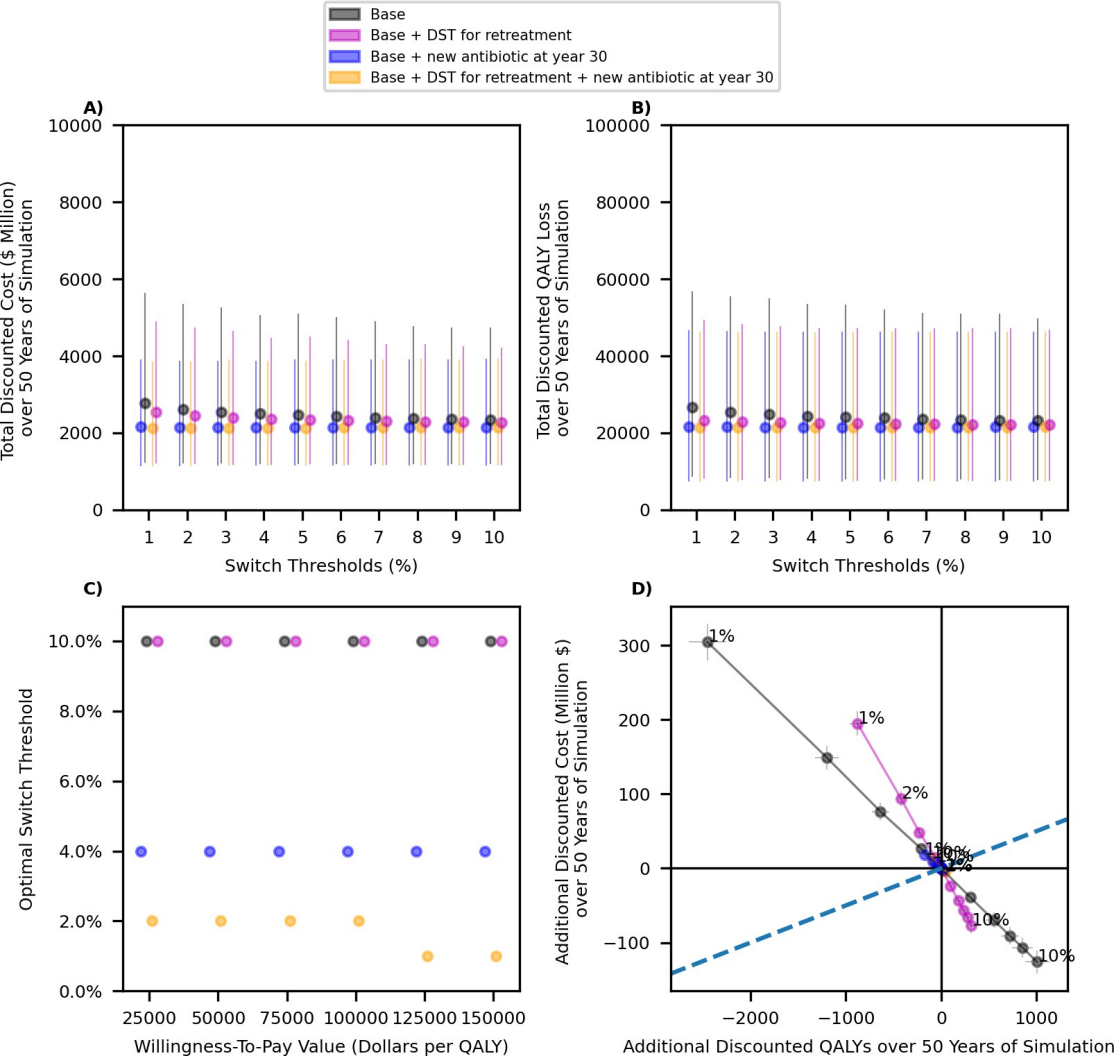
**The impact of changing the switch threshold on the total discounted cost and QALY loss over 50 years of simulation (panels A and B), and identifying the optimal switch threshold for each scenario (panels C and D).** Dots represent the mean value. Bars represent the 95% uncertainty intervals in panels A–C represent confidence intervals in panel D. Dollars are referring to 2022 US$. Panel C displays threshold that minimizes the loss in the population NHB. In the cost-effectiveness plane displayed in panel D, the origin for each scenario represents the same scenario when the 5% switch threshold is selected (hence, the x- and y-axis represent the change in QALYs and cost for each scenario as the switch threshold changes). The dashed line represents the WTP threshold of $50,000 per QALY. DST, drug-susceptibility testing; NHB, net health benefit; QALY, quality-adjusted life-years; WTP, willingness-to-pay.

The choice of the switch threshold does not have a significant impact on the total discounted costs and QALY loss over 50 years of simulation for scenarios considered here. However, the introduction of a new antibiotic at year 30 would reduce the total costs and QALY loss due to gonorrhea (Fig 4A and 4B), and access to DSTs to inform the selection of retreatment antibiotics could slightly reduce the cost and QALY loss (Fig 4A and 4B). Similar behavior is observed for scenarios with higher cost of Drug B (panels A and B of Fig D in S1 Appendix), higher probability that additional resistance emerges under treatment (panels A and B of Fig G in S1 Appendix), and lower transmissibility of resistant strains (panels A and B of Fig J in S1 Appendix).

The optimal switch threshold (i.e., the threshold that minimizes the loss in the population NMB) varies by the availability and cost of future antibiotics, the availability of DSTs to inform retreatment antibiotics (Fig 4C). A lower threshold (e.g., 1% to 2%, 4%) is optimal when a new antibiotic is expected to become available at year 30 (comparing black to blue dots in Fig 4C) or when DSTs are available to inform retreatment antibiotics (comparing orange to blue dots in Fig 4C). In contrast, if the cost of future antibiotics is expected to increase, a higher switch threshold may need to be selected depending on the decision-maker’s WTP value (comparing panel C in Fig 4 and Fig D in S1 Appendix). When a new antibiotic is expected to become available in the future, deviating from the optimal threshold has less severe health and cost consequences; changing the switch threshold in these scenarios does not markedly change the cost and QALYs (Fig 4A, 4B and 4D).

**Fig 5:**
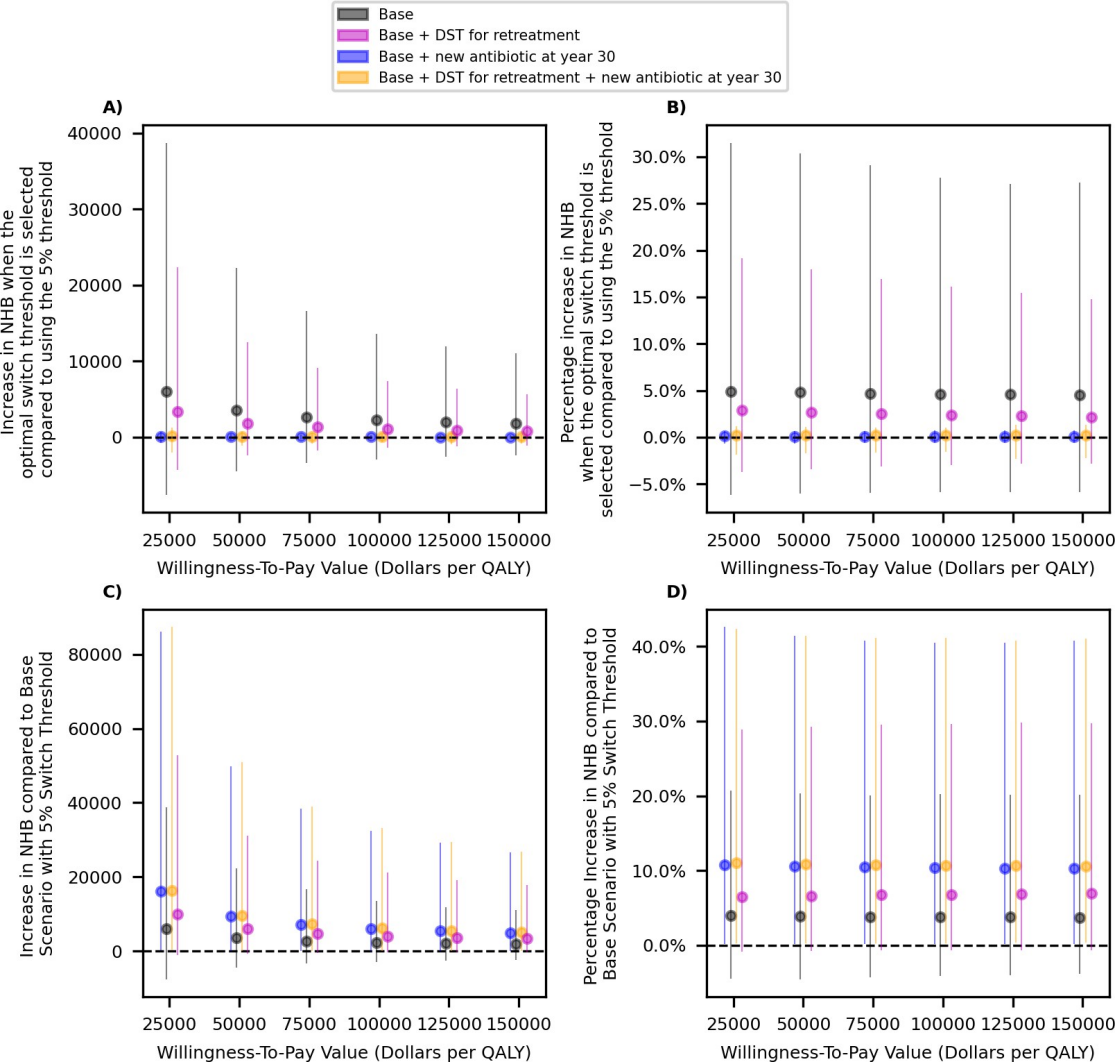
**The impact of optimizing the switch threshold (panels A and B) and the impact of scenarios for the availability of new antibiotics in the future (panels C and D) on the population NHB.** Dots represent the mean value. Bars represent the 95% uncertainty intervals. Dollars are referring to 2022 US$. In panels A and B, estimate for each scenario are provided with respect to the same scenario but when the 5% switch threshold is used. In panels C and D, estimates for each scenario are provided with respect to the “Base Scenario” where only Drugs A and B are available for first-line therapy of gonorrhea over the next 50 years and the switch threshold of 5% is selected. DST, drug-susceptibility testing; NHB, net health benefit; QALY, quality-adjusted life-years.

This is also reflected in Fig 5A and 5B, which demonstrate that the gain in the population NHB resulted from optimizing the switch threshold is relatively small under all scenarios considered here, but selecting the optimal threshold is more important if the availability of future antibiotics is limited (comparing black and purple dots to others in Fig 5A and 5B). In contrast, the introduction of a new antibiotic in the future is expected to result in a larger gain in the population NMB (Fig 5C and 5D). The availability of DST to inform retreatment antibiotic slightly improves the population NMB under each scenario of antibiotic availability (Fig 5C and 5D). These conclusions are robust to the assumptions related to the cost of Drug B (Fig E in S1 Appendix), probability that additional resistance emerges under treatment (Fig H in S1 Appendix), and the transmissibility of resistant strains (Fig K in S1 Appendix).

## Discussion

The antibiotics included in the guidelines for the empiric treatment of gonorrhea are recommended to change when the resistance prevalence exceeds 5% [8,9]. Using a transmission model of gonococcal infection among the MSM population in the US, we projected how changing this switch threshold would impact the overall cost and loss in QALYs associated with gonorrhea. Under the scenario where 3 antibiotics are available over the next 50 years (2 suitable for the first-line therapy of gonorrhea and 1 suitable only for the retreatment of resistant infections), changing the switch threshold between 1% and 10% does not meaningfully impact the annual number of gonorrhea cases, total costs or total QALY losses associated with gonorrhea. Our analysis, however, suggests that the switch threshold that maximizes the population NHB depends on the availability of future antibiotics, the cost of future antibiotics, and access to DSTs to inform the retreatment antibiotic. When a new antibiotic is expected to become available in the future, a lower threshold would lead to a higher population NHB, compared to using the 5% threshold. Reducing the switch threshold increases the probability that standardized regimen matches the susceptibility profile of a gonococcal infection, which improves treatment outcomes and reduces the secondary transmission of infection. However, it also shortens the lifespan of first-line antibiotics as it leads to switching to newer antibiotics at a faster pace, which could result in emergence and spread of additional resistant gonococcal strains. The introduction of a new antibiotic minimizes these adverse consequences.

Our analysis also indicates that if DSTs are available to identify retreatment antibiotics when first-line therapy is not successful, selecting a lower switch threshold would result in higher population NHB (Fig 4D). DSTs allow customizing the treatment regimen according to the antibiotic susceptibility of an individual infection. As a substantial portion of infections may be susceptible to older antibiotics (e.g., to Drug A when Drug B is the recommended first-line therapy), DSTs allow to prescribe older antibiotics, which alleviates the selective pressure for the development of resistance to newer antibiotics [13,23,24].

Our analysis also suggests that the gain in NHB from optimizing the switch threshold is relatively small (Fig 5A and 5B, panels A and B of Fig E in S1 Appendix, panels A and B of Fig H in S1 Appendix, and panels A and B of Fig K in S1 Appendix). This is because under the scenarios of antibiotic and DST availability considered here, changing the switch threshold does not have a meaningful impact on outcomes such as rate of gonorrhea cases or treatment failure, that determine the overall cost and QALYs loss associated with gonorrhea (Fig 3A and 3B). In contrast, an introduction of a new antibiotic that is suitable for first-line therapy of gonorrhea over the next 30 years is expected to substantially improve these outcomes and the population NHB (Fig 5C and 5D, Figs E, H, and K in S1 Appendix).

Our study has several limitations. First, our model describes the spread of *N*. *gonorrhoeae* only among MSM. Compared to heterosexual men and women, the prevalence of gonorrhea and AMR gonorrhea is particularly high among MSM [12,25]. Therefore, the benefits of optimizing the switch threshold or the gain from regular introduction of new antibiotics might be lower for populations with lower burden of gonorrhea and AMR gonorrhea. Second, as suggested by our results, the impact of a switch threshold on the overall cost and burden of gonorrhea depends on control measures that would become available in the future. We considered scenarios related to the availability of new antibiotics and DSTs for retreatment of cases. We, however, noted that other future scenarios could also be considered including scenarios related to the availability of vaccine or common use of rapid DSTs to inform first-line therapy. In addition, the increased use of doxycycline post-exposure prophylaxis (Doxy-PEP) could impact the burden of gonorrhea and AMR gonorrhea [26]. Studies suggest that the potential impact of Doxy-PEP on gonorrhea prevalence is likely short term and depends on the level of doxycycline resistance in the population [27]. Moreover, use of doxycycline may lead to an increase in resistance overall, not just to doxycycline, but to other drugs, because of linked resistance [28]. As limited data are available on the uptake of these innovations and their impact on sexual behavior and the burden of gonorrhea and AMR gonorrhea, we did not consider them in our modeling analysis. Third, we assumed that treatment guidelines are updated when the resistance prevalence reaches a prespecified threshold. In practice, policymakers may not necessarily wait until the selected threshold is reached to revise the treatment guidelines. For example, the decision to abandon the use of the oral extended spectrum cephalosporin cefixime as first-line therapy was made before the resistance to this drug reaches the 5% resistance and it was based on the observed upward trajectory of resistance [29]. Fourth, since our simulation was a compartmental model, it did not include certain complexities related to the spread and the control of sexually transmitted diseases such as the treatment of sex partners when a partner is diagnosed with gonorrhea. Finally, while we assumed that only 1 antibiotic is prescribed for first-line therapy, dual therapy has also been occasionally recommended. For example, between 2010 and 2020, dual therapy with ceftriaxone and azithromycin were recommended for the first-line treatment of gonorrhea in the US [5,30]. Fifth, antibiotics for gonorrhea treatment might also be used for other infections and their use might have impact on other infections and microbiome. We did not account for these indirect impacts in estimating the cost and QALYs associated with different scenarios considered in our analysis.

The main strength of our study is the use of a calibrated simulation model of gonococcal infection to project the long-term population-level cost and health outcomes under different health strategies and future scenarios. This allows us to investigate how changing the resistance switch threshold and the cost and the availability of novel antibiotics are expected to impact the health and financial burden of gonorrhea and AMR gonorrhea in the long term.

In conclusion, changing the resistance threshold that determines first-line therapy is not expected to substantially impact the overall cost and QALYs associated with gonorrhea. Nevertheless, the optimal switch threshold depends on the availability of future antibiotics and DSTs for informing regimen for the retreatment of infections that do not respond to first-line therapy. Our findings highlight the importance of regular, dependable development of new antibiotics, and in such scenarios where the future pipeline of antibiotics is reasonably certain, lowering the switch threshold would result in higher population NHB. As our analysis relied on a simulation model of AMR gonorrhea among the US MSM population, additional research is required to evaluate the generalizability of our findings to settings outside the US and to non-MSM population. Future studies could also investigate the impact of choosing the switch thresholds based on the population-level risk of AMR gonococcal infection, which could vary over geographic regions and/or population groups [31,32]. This allows for making newer antibiotics available to populations at higher risk of AMR gonorrhea while curbing its use to minimize the risk for the emergence of new resistant strains.

## Supporting information

S1 Checklist CHEERS 2022 Checklist. (PDF)

S1 Appendix Additional model details and results of sensitivity analyses. Table A. Model notation. Table B. Transitions between model compartments when a new drug (Drug C) becomes available in addition to Drugs A and B. Table C. Prior and Posterior distribution of parameters. Fig A. The prevalence of infection and the spread of resistance in different sexual activity group for trajectories displayed in Fig 2. Fig B. Posterior distribution of model parameters listed in Table C in S1 Appendix. Fig C. Probability tree for infected individuals without or fail the treatment. Fig D. The impact of changing the switch threshold on the total discounted cost and QALY loss over 50 years of simulation (panels A and B), and identifying the optimal switch threshold (panels C and D), when the cost of Drug B is twice the cost Drug A. Fig E. The impact of optimizing the switch threshold (panels A and B) and the scenarios for the availability of new antibiotics in the future (panels C and D) on the population net health benefit (NHB) when the cost of Drug B is twice the cost of Drug A. Fig F. The impact of changing the switch threshold on the average annual rate of gonorrhea cases (panel A), average annual rate of treatment failure (panel B), the average annual rate of drug-susceptibility testing (DST) (panel C), and the proportion of cases treated with Drug A, Drug B, and Drug M (panels D–F), when the probability that resistance develops during treatment is increased to 10^[−5,−3]^. Fig G. The impact of changing the switch threshold on the total discounted cost and QALY loss over 50 years of simulation (panels A and B), and identifying the optimal switch threshold for each scenario (panels C and D) when the probability that resistance develops during treatment is increased to 10^[−5,−3]^. Fig H. The impact of optimizing the switch threshold (panels A and B) and the scenarios for the availability of new antibiotics in the future (panels C and D) on the population net health benefit (NHB) when the probability that resistance develops during treatment is increased to 10^[−5,−3]^. Fig I. The impact of changing the switch threshold on the average annual rate of gonorrhea cases (panel A), average annual rate of treatment failure (panel B), the average annual rate of drug-susceptibility testing (DST) (panel C), and the proportion of cases treated with Drug A, Drug B, and Drug M (panels D–F) when the relative transmissibility of resistant strains is [0.6, 1]. Fig J. The impact of changing the switch threshold on the total discounted cost and QALY loss over 50 years of simulation (panels A and B), and identifying the optimal switch threshold for each scenario (panels C and D) when the relative transmissibility of resistant strains is [0.6, 1]. Fig K. The impact of optimizing the switch threshold (panels A and B) and the scenarios for the availability of new antibiotics in the future (panels C and D) on the population net health benefit (NHB) when the relative transmissibility of resistant strains is [0.6, 1].
(PDF)

## Abbreviations

AMR: antimicrobial-resistant
DGI: disseminated gonococcal infection
Doxy-PEP: doxycycline post-exposure prophylaxis
DST: drug-susceptibility testing
GISP: Gonococcal Isolate Surveillance Project
MSM: men who have sex with men
NHB: net health benefit
PHC: Personal Health Care
QALY: quality-adjusted life-years
WTP: willingness-to-pay
