# Supplementary material for: Assessing thresholds of resistance prevalence at which empiric treatment of gonorrhea should change among men who have sex with men in the US: A cost-effectiveness analysis
Source: PLoS Med. 2024 Jul 8;21(7):e1004424. doi: 10.1371/journal.pmed.1004424 (PMC11262662; doi:10.1371/journal.pmed.1004424)
Supplement: S1 Appendix — Table A. Model notation. Table B. Transitions between model compartments when a new drug (Drug C) becomes available in addition to Drugs A and B. Table C. Prior and Posterior distribution of parameters. Fig A. The prevalence of infection and the spread of resistance in different sexual activity group for trajectories displayed in Fig 2. Fig B. Posterior distribution of model parameters listed in Table C in S1 Appendix. Fig C. Probability tree for infected individuals without or fail the treatment. Fig D. The impact of changing the switch threshold on the total discounted cost and QALY loss over 50 years of simulation (panels A and B), and identifying the optimal switch threshold (panels C and D), when the cost of Drug B is twice the cost Drug A. Fig E. The impact of optimizing the switch threshold (panels A and B) and the scenarios for the availability of new antibiotics in the future (panels C and D) on the population net health benefit (NHB) when the cost of Drug B is twice the cost of Drug A. Fig F. The impact of changing the switch threshold on the average annual rate of gonorrhea cases (panel A), average annual rate of treatment failure (panel B), the average annual rate of drug-susceptibility testing (DST) (panel C), and the proportion of cases treated with Drug A, Drug B, and Drug M (panels D–F), when the probability that resistance develops during treatment is increased to 10[−5,−3]. Fig G. The impact of changing the switch threshold on the total discounted cost and QALY loss over 50 years of simulation (panels A and B), and identifying the optimal switch threshold for each scenario (panels C and D) when the probability that resistance develops during treatment is increased to 10[−5,−3]. Fig H. The impact of optimizing the switch threshold (panels A and B) and the scenarios for the availability of new antibiotics in the future (panels C and D) on the population net health benefit (NHB) when the probability that resistance develops during treatment is increas [file pmed.1004424.s002.pdf]

Additional model details and results of sensitivity analyses

Additional model details

We developed a stochastic compartmental model to simulate the transmission of gonorrhea among the MSM population. To estimate the size of MSM aged 14 years and older, we used the results of a meta-analysis of US population-based surveys that estimated the proportion of MSM among males 13 and older at 3.9% [1]. According to the 2015 U.S. Census, the size of the male population of age 14 and older is 125,092,000. Therefore, we estimated the MSM population of age 14 and older at 4,878,588.

We divided the model population into three groups of sexual activity (low, intermediate, and high, indexed by $k \in \{1,2,3\}$), based on annual rates of partner change. We assumed that each group has population $N_k(t)$, and that individuals remained in a given sexual-activity group for the duration of their sexual lifespan. The rate of partner change (c_{min}) in the low activity group and the relative rate of partner change (r_k) in the different risk groups were estimated by model fitting. The rate of partner change for each activity group could be represented by (with $r_1 = 1$):

$$c_k = r_k * c_{min}$$

We used the approach of Garnett et al [2]. to derive the probability of contact within and between groups, with the parameter ϵ (range from 0 to 1) describing mixing between groups. The probability that a person of sexual-activity group k formed a partnership with a person of sexual-activity group k' is calculated as:

$$p_{kk'}(t) = \epsilon\delta_{kk'} + (1-\epsilon)\frac{c'_k N_{k'}(t)}{\sum_{k'=1}^{3} c'_k N_{k'}(t)}$$

where $\delta_{kk'} = 1$ if $k = k'$ and 0 otherwise. The rate at which susceptible individuals are infected from partners in group k' depends on the partner change rate (c_k), the transmission probability per partnership (β_0), and the proportion of sexual partnerships occurring between sexual activity groups k and k' ($p_{kk'}$):

$$\beta_{kk'}(t) = \beta_0 c_k p_{kk'}(t)$$

The rate at which susceptible individuals are infected with the resistance profile $i \in \{0, A, B, AB\}$ is calculated as:

$$\mathcal{F}_{ki}(t) = \sum_{k'=1}^{3} \frac{\beta_{kk'}(t)\gamma_i(\hat{I}_{k'i}(t) + I_{k'i}(t))}{N_{k'}(t)}$$

where $0 \leq \gamma_i \leq 1$ is the fitness cost associated with resistance profile $i \in \{A, B, AB\}$ (with $\gamma_0 = 1$) and are determined through calibration.

To simulate the model, we sampled from the multinomial distribution associated with each compartment and then use these realizations to identify the new epidemic state at the next time step. For example, for each sex activity group k, consider a particular compartment S in which members depart due to m events each of which is occurring at the rate $\mu_{km}, m \in \{1,2,\ldots,M\}$. If the number of individuals in compartment S at time t is $S(t)$, then the number of individuals that leave this compartment due to events $m \in \{1,2,\ldots,M\}$ follows a multinomial

distribution with total counts of $S(t)$ and probabilities $(p_{k0}, p_{k1}, p_{k2}, \dots, p_{kJ})$, where $p_{k0} = 1 - e^{-\sum_{m\in M}\mu_{km}\Delta t}$ is the probability of not leaving the compartment S during $[t, t + \Delta t]$, and $p_{km} = \frac{\mu_{km}}{\sum_{m\in M}\mu_{km}}(1 - e^{-\sum_{m\in M}\mu_{km}\Delta t})$ is the probability of leaving the compartment S during $[t, t + \Delta t]$ due to the event $m \in \{1,2, \dots, M\}$. When compartment Z represents susceptible group, the event *m* will be the same with the resistance profile i, so that the rate $\mu_{km} = \mathcal{F}_{ki}$.

The simulation of compartment flows for each sexual activity group k is shown in Fig 1. Before switching the antibiotic, the infected individuals move to treatment compartments via red arrows in Fig 1. After switching the antibiotic, the infected individuals move to treatment compartments via green arrows in Fig 1. Other transitions between compartments remain the same.

Modeling the introduction of a new antibiotic

When the prevalence of resistance to Drug B passes the selected switch threshold, a new antibiotic (Drug C, if available) will replace Drug B in first-line therapy. Since our model includes only two first-line antibiotics, the introduction of the new Drug C requires redefining Drugs A and/or B, such that Drug A now represents the previous first-line antibiotic (i.e., Drug B) and Drug B now represents the new Drug C (which is used as second-line therapy). This is implemented by moving the members of model compartments according to the rules described in Table B.

Model calibration

The model is calibrated against estimates of gonorrhea prevalence (4.5% [3.6%, 5.4%] among MSM)[3], the annual gonorrhea rate in 2018 (6,508 cases per 100,000 MSM)[4], and the proportion of gonorrhea cases with symptoms (67.9% [64.4-71.4%])[5]. Our calibration procedures are based on the use of common random numbers to simulate epidemic trajectories, that allow us to reproduce a simulated trajectory by knowing the random number seed. For each simulation trajectory, we approximate the likelihood of observations using a pseudolikelihood function that consists of three components as described below.

Component 1: Likelihood of gonorrhea prevalence

To calculate the likelihood of observing a prevalence $\hat{s}/\hat{S}$ in year t if a given simulated trajectory represents the reality, we assumed that $\hat{s}$ (number of individuals diagnosed with gonorrhea) follows a binomial distribution with $\hat{S}$ trials (total number of individuals tested) and the success probability τ_t, where τ_t is the prevalence of gonorrhea in year t of the simulation:

$$L_1 = \prod_{t=1}^{10} \binom{\hat{S}}{\hat{s}} \tau_t^{\hat{s}} (1 - \tau_t)^{\hat{S}-\hat{s}}.$$

We used $\hat{S}$ =2,075 and $\hat{s}$=93 (resulting in the estimated prevalence of 4.5%), as reported by Jones et al [3].

Component 2: Likelihood of annual rate of reported gonorrhea cases

To calculate the likelihood of observing a corresponding cases rate in year t if a given simulated trajectory represents the reality, we assumed that the 2018 estimate of 6,508 cases of gonorrhea per 100,000 MSM is calculated as $\hat{h}/\hat{H} \times 100{,}000$, where $\hat{h}$ is the number of gonorrhea cases observed in a sample MSM population

of size $\widehat{H}$. We assumed that $\hat{h}$ follows a binomial distribution with $\widehat{H}$ trials and success probability ρ_t, where ρ_t is the proportion of the simulated population year t that got diagnosed with gonorrhea:

$$L_2 = \sum_{t=1}^{10} \binom{\widehat{H}}{\hat{h}} \rho_t^{\hat{h}} (1 - \rho_t)^{\widehat{H} - \hat{h}}.$$

We assumed that the estimated 6,508 cases of gonorrhea per 100,000 MSM is with 20% error (equivalent to having a reported confidence interval of [5,206 – 7,810]) since no confidence intervals were reported for this estimate. $\widehat{H}$ is approximated by the half-length of the confidence interval for the estimated annual rate of reported gonorrhea cases:

$$HL = 100{,}000 \times z_{\alpha/2} \sqrt{\frac{\mu(1-\mu)}{\widehat{H}}},$$

where $\mu = \hat{h}/\widehat{H}$ and $z_\alpha/2$ is the upper $\alpha/2$ critical point for the standard normal distribution. We obtained $\widehat{H} = 1{,}381$, when plugging in the values $HL = (7{,}810 - 5{,}206)/2 = 1{,}302$, α=0.05, and $\hat{h}/\widehat{H} = 0.06508$.

Component 3: Likelihood of proportion of gonorrhea cases that are symptomatic

To calculate the likelihood of observing a corresponding proportion of symptomatic gonorrhea cases in year t if a given simulated trajectory represents the reality, we assumed that $\hat{r}$, which is the estimated number of symptomatic cases, follows a binomial distribution. There are $\hat{R}$ trials and the success probability is θ_t, which is the proportion of gonorrhea cases in year t of the simulation that are symptomatic:

$$L_3 = \sum_{t=1}^{10} \binom{\hat{R}}{\hat{r}} \theta_t^{\hat{r}} (1 - \theta_t)^{\hat{R} - \hat{r}}.$$

We used $\hat{r} = 466$ and $\hat{R} = 686$ based on the results reported by Newman et al [5].

Total pseudolikelihood

The total likelihood of a given simulation trajectory is obtained by adding the natural logarithm of each likelihood of observation:

$$\ln \mathcal{L} = ln\, L_1 + ln\, L_2 + ln\, L_3.$$

To improve the efficiency of the calibration procedure, we halted the simulation of a trajectory if any of the conditions below occurs:

1. Gonorrhea prevalence falls out of the range [1%, 30%].
2. Annual rate of reported gonorrhea cases falls out of the range [1,000, 20,000],
3. Annual percentage of detected gonorrhea cases that are symptomatic less than 50%.
4. Gonorrhea prevalence among the low sexual activity group falls out of the range [0%, 4%].
5. Gonorrhea prevalence among the medium sexual activity group falls out of the range [2%, 8%].
6. Gonorrhea prevalence among the high sexual activity group falls out of the range [5%, 25%].

To make sure that the resistance to Drugs A and B emerges during the simulation horizon (50 years), we eliminated trajectories where the prevalence of resistance to Drug A or Drug B never reached 5%. This is consistent with historical data as the prevalence of resistance to antibiotics that were previously used to treat gonorrhea has reached this threshold.

Projections and parameter estimation

We simulated enough trajectories to obtain 3,000 trajectories that did not violate the feasibility conditions defined above. For each of the simulation trajectory, we randomly generate parameters' values from the probability distributions presented in Table C. These prior distributions are obtained from existing scientific literature when such estimates are available. When the prior distributions are not available, we assume they have biologically-feasible distributions. We calculated the weight of each pseudolikelihood $\ln \mathcal{L}_i$ for the simulation trajectory $i \in \{1,2,\ldots,N_0\}$ as:

$$w_i = \frac{e^{ln\mathcal{L}_i - L_{max}}}{\sum_{j=1}^{N_0} e^{ln\mathcal{L}_i - L_{max}}},$$

where L_{max}=max{$\ln \mathcal{L}_1, \ln \mathcal{L}_2, \ldots, \ln \mathcal{L}_{N_0}$}. We next draw 1000 trajectories based on likelihood weights w_i with replacement. We calculated the mean and 95% posterior intervals of model parameters (Table C) using the parameter values associated with the 200 trajectories (out of the accepted 1,000 trajectories). The posterior distributions are also listed in Table C. Fig B shows the histograms of resampled parameter values after calibration.

Cost and disutility parameters

To project the overall cost and QALY loss under scenarios considered in this analysis, we included costs that are incurred due to diagnosis, treatment, and drug susceptibility testing (Table 1), and QALY loss that are due to gonorrhea symptoms and treatment side effect (Table 2). We also assumed that a small portion of individuals who receive an ineffective treatment or recover without treatment may develop sequalae. We estimated the cost and QALY loss due to sequalae according to the probability tree displayed in Fig C.

We further assumed that eligibility and frequency of screening for gonococcal infection remain the same under strategies considered here. Therefore, we did not include costs related to screening since they are expected to be the same under all scenarios modelled here.

Sensitivity analysis

In addition to the results in the manuscript, we performed the sensitivity analysis by increasing the cost of Drug B twice as much as Drug A, increase the probability of developing resistance during the treatment, and increase the uncertainty of the relative transmissibility of the resistant strains. The sensitivity analysis results are presented from Fig D to Fig K.

Tables

Table A. Model notation

Parameter	Description
$S(t)$	Number of susceptible individuals at time t
$\hat{I}_i(t)$	Number of asymptomatic infections with resistance profile i at time t
	$\hat{I}_0(t), \hat{I}_A(t), \hat{I}_B(t), \hat{I}_{AB}(t)$
$I_i(t)$	Number of symptomatic infections with resistance profile i at time t
	$I_0(t), I_A(t), I_B(t), I_{AB}(t)$
$\hat{I}_i \mid Tx\text{-}j(t)$	Number of asymptomatic infections with resistance profile i under treatment with drug $j \in \{A, B\}$
	$\hat{I}_0, \hat{I}_A, \hat{I}_B, \hat{I}_{AB} \mid Tx\text{-}A(t)$
	$\hat{I}_0, \hat{I}_A, \hat{I}_B, \hat{I}_{AB} \mid Tx\text{-}B(t)$
$I_i \mid Tx\text{-}j(t)$	Number of symptomatic infections with resistance profile i under treatment with drug $j \in \{A, B, C\}$
	$I_0, I_A, I_B, I_{AB} \mid Tx\text{-}A(t)$
	$I_0, I_A, I_B, I_{AB} \mid Tx\text{-}B(t)$
$I_i \mid F_j(t)$	Number of infections with resistance profile i that fail the treatment with drug $j \in \{A, B, C\}$
	$I_A, I_{AB} \mid F_{Tx\text{-}A}(t)$
	$I_B, I_{AB} \mid F_{Tx\text{-}B}(t)$
	$I_{AB} \mid F_{Tx\text{-}C}(t)$

Table B. Transitions between model compartments when a new drug (Drug C) becomes available in addition to Drugs A and B. Note that, once Drug C becomes available, the Drug A in the model represents the old first-line antibiotic (i.e., Drug B) and Drug B in the model represents the new second-line antibiotic (i.e., Drug C).

Transition	Explanation
For compartments representing infection:	
I_0 stays	Infections susceptible to the old A and B remain susceptible to the new A and B.
$\hat{I}_0$ stays	Infections susceptible to the old A and B remain susceptible to the new A and B.
$I_A \rightarrow I_0$	Infections resistant to the old A are now susceptible to the new A.
$\hat{I}_A \rightarrow \hat{I}_0$	Infections resistant to the old A are now susceptible to the new A.
$I_B \rightarrow I_A$	Infections resistant to the old B are now resistant to the new A.
$\hat{I}_B \rightarrow \hat{I}_A$	Infections resistant to the old B are now resistant to the new A.
$I_{AB} \rightarrow I_A$	Infections resistant to both old A and B are now resistant to only the new A.
$\hat{I}_{AB} \rightarrow \hat{I}_A$	Infections resistant to both old A and B are now resistant to only the new A.
For compartments representing treatment with Drug A:	
$I_0 \mid Tx\text{-}A$ stays	The new A is effective for I_0.
$\hat{I}_0 \mid Tx\text{-}A$ stays	The new A is effective for I_0.
$I_A \mid Tx\text{-}A \rightarrow I_0 \mid Tx\text{-}A$	Those receiving the old A now receive the old second-line drug (i.e., the new A).
$\hat{I}_A \mid Tx\text{-}A \rightarrow \hat{I}_0 \mid Tx\text{-}A$	Those receiving the old A now receive the old second-line drug (i.e., the new A).
$I_B \mid Tx\text{-}A$ stays	The old A was effective for I_B.
$\hat{I}_B \mid Tx\text{-}A$ stays	The old A was effective for I_B.
$I_{AB} \mid Tx\text{-}A \rightarrow I_A \mid Tx\text{-}A$	The old A was ineffective for I_{AB}.
$\hat{I}_{AB} \mid Tx\text{-}A \rightarrow \hat{I}_A \mid Tx\text{-}A$	The old A was ineffective for I_{AB}.
For compartments representing treatment with Drug B:	
$I_0 \mid Tx\text{-}B \rightarrow I_0 \mid Tx\text{-}A$	The new A represents the old B.
$\hat{I}_0 \mid Tx\text{-}B \rightarrow \hat{I}_0 \mid Tx\text{-}A$	The new A represents the old B.
$I_A \mid Tx\text{-}B \rightarrow I_0 \mid Tx\text{-}A$	Infections resistant to the old A are now susceptible to the new A.
$\hat{I}_A \mid Tx\text{-}B \rightarrow \hat{I}_0 \mid Tx\text{-}A$	Infections resistant to the old A are now susceptible to the new A.
$I_B \mid Tx\text{-}B \rightarrow I_A \mid Tx\text{-}A$	Infections resistant to the old B are now resistant to the new A.
$\hat{I}_B \mid Tx\text{-}B \rightarrow \hat{I}_A \mid Tx\text{-}A$	Infections resistant to the old B are now resistant to the new A.
$I_{AB} \mid Tx\text{-}B \rightarrow I_A \mid Tx\text{-}A$	The old B was ineffective for I_{AB}.
$\hat{I}_{AB} \mid Tx\text{-}B \rightarrow \hat{I}_A \mid Tx\text{-}A$	The old B was ineffective for I_{AB}.
For compartments representing treatment failure after receiving Drug A or Drug B:	
$I_A \mid F_{Tx\text{-}A} \rightarrow I_0 \mid Tx\text{-}A$	The new A is now effective for infection that failed the old A
$I_{AB} \mid F_{Tx\text{-}A} \rightarrow I_A \mid Tx\text{-}A$	The new A is not effective for infections resistant to both old A and B.
$I_B \mid F_{Tx\text{-}B} \rightarrow I_A \mid F_{Tx\text{-}A}$	Infections resistant to old B are now resistant to new A and new A represents old B
$I_{AB} \mid F_{Tx\text{-}B} \rightarrow I_A \mid F_{Tx\text{-}A}$	Infections resistant to old B are now resistant to new A and new A represents old B

Table C. Prior and Posterior distribution of parameters

Parameter	Prior Distribution (All Uniform)	Mean and 95% Posterior Interval	Sources to Inform Prior Distribution
Transmission parameter (β_0) (1/year)	[0.01, 0.7]	0.18 [0.09, 0.45]	Assumption based on [1]
Probability of developing resistance while receiving Drug A	$10^{[-6,-4]}$	$10^{[-4.31]}$; $10^{[-5.44,-4.01]}$	Assumption based on [1]
Probability of developing resistance while receiving Drug B	$10^{[-6,-4]}$	$10^{[-4.25]}$; $10^{[-5.37,-4.01]}$	Assumption based on [1]
Probability of symptomatic infection (α_0)	[0.1, 0.9]	0.50 [0.28, 0.65]	Assumption based on [6-8]
Proportion of infections that were symptomatic at time 0 of simulation (α_1)	[0.010, 0.025]	0.018 [0.010, 0.024]	Assumption based on [1]
Initial prevalence of infection in different sexual-activity groups			Assumption based on [7]
Low	[0.004, 0.014]	0.009 [0.005, 0.012]	
Intermediate	[0.02, 0.07]	0.045 [0.024, 0.061]	
High	[0.08, 0.28]	0.180 [0.096, 0.244]	
Proportion of the population in			Assumption based on [7]
low sexual activity group		0.3	
intermediate sexual activity group		0.6	
high sexual activity group		0.1	
Time until recovery without treatment (1/months)	[1, 60]	29.6 [4.2, 56.6]	Assumption based on [6]
Time until getting screened for infection (1/years)	[0.5, 5]	2.5 [1.6, 3.5]	Assumption based on [6, 7]
Time until seeking treatment for a symptomatic infection (1/days)	[1, 14]	7.7 [1.9, 13.6]	Assumption based on [5, 6, 9]
Time until retreatment (1/days)	[1, 14]	7.3 [1.5, 13.0]	Assumption based on [5, 6]
Treatment duration for Drugs A and B (1/days)	[1, 2]	1.5 [1.1, 2.0]	Assumption based on [1]
Relative transmissibility of resistant strains			Assumption based on [1]
γ_A	[0.9, 1]	0.91 [0.81, 1.00]	
γ_B	[0.9, 1]	0.90 [0.81, 1.00]	
γ_{AB}	$0.45(\gamma_A + \gamma_B)$	0.81 [0.75, 0.88]	
Annual rate of partner change in low sexual activity group per year (c_{min})	[0.75, 1.25]	0.94 [0.73, 1.20]	Assumption based on [10]
Relative rate of partner change in sexual activity groups			Assumption based on [10]
Low		1	
Intermediate	[4,10]	7.29 [4.36, 9.96]	
High	[19, 25]	21.82[19.26, 24.85]	
Mixing parameter (ϵ)	[0, 1]	0.27 [0.01, 0.62]	Assumption

Figures

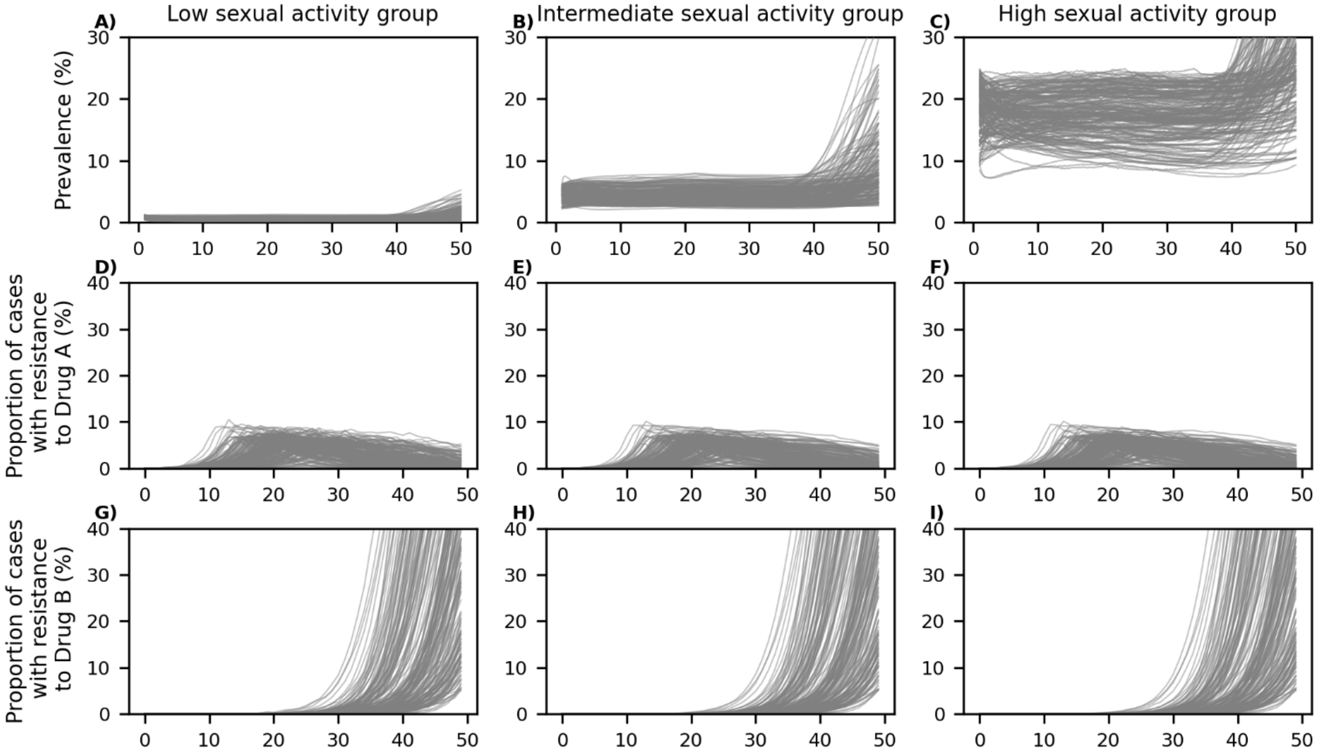

Fig A: The prevalence of infection and the spread of resistance in different sexual-activity group for trajectories displayed in Fig 2. See the caption Fig 2 for additional details.

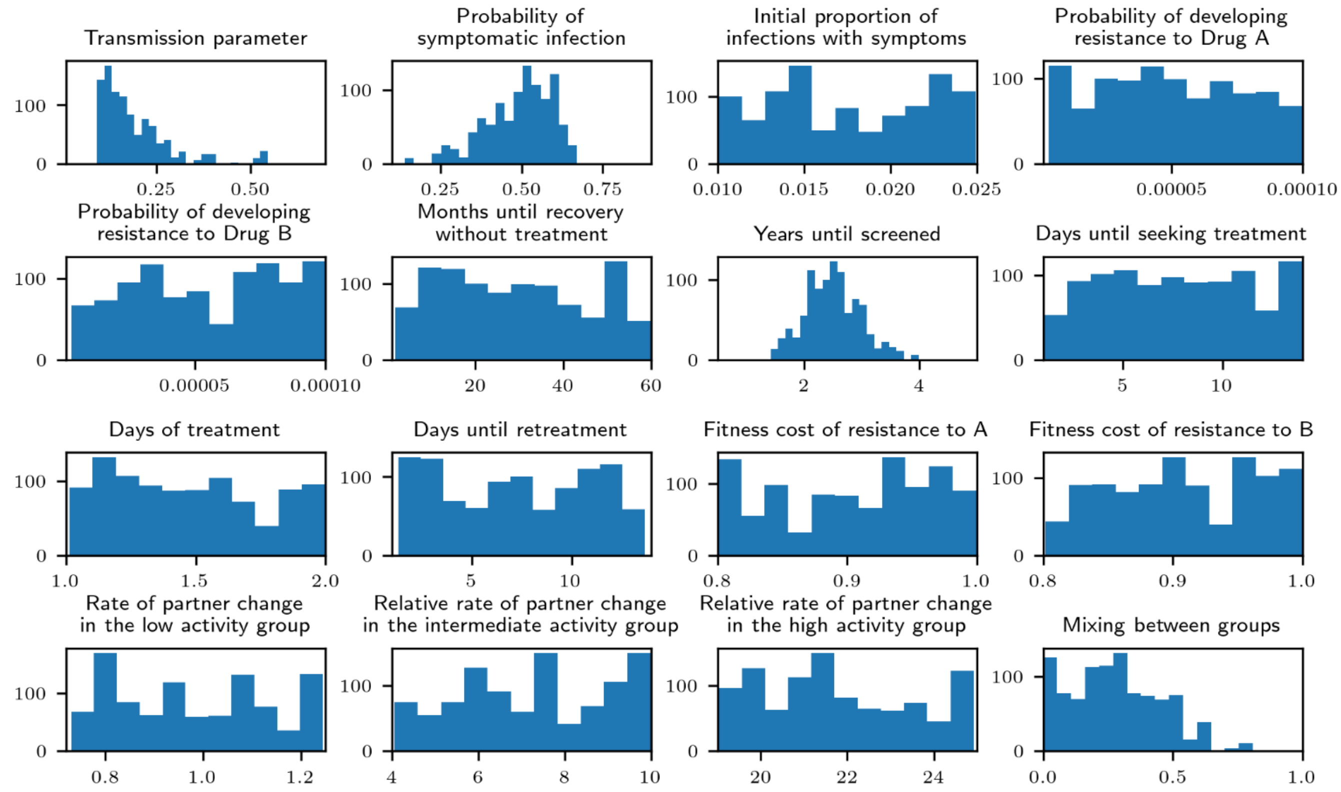

Fig B: Posterior distribution of model parameters listed in Table C. The range of horizontal axis on each subplot represents the prior distribution of the parameter.

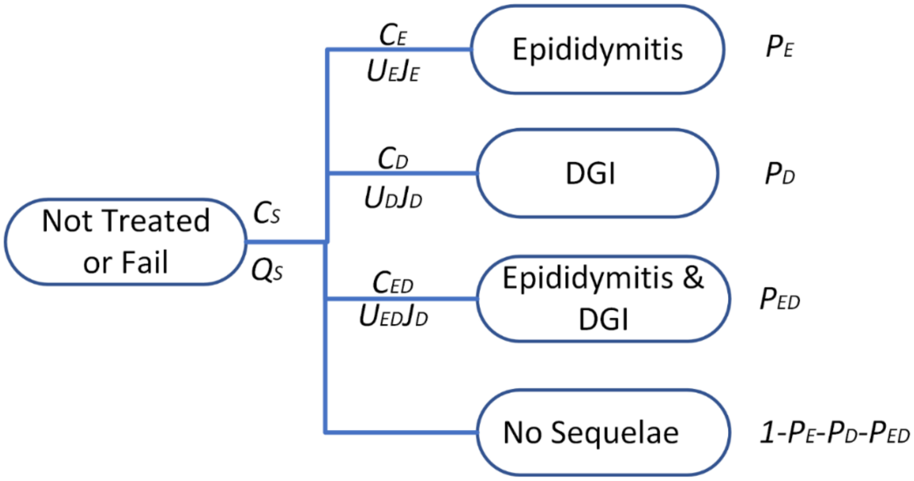

Fig C: Probability tree for infected individuals without or fail the treatment. The parameters of this probability tree are listed in Table 1. DGI: Disseminated Gonococcal Infection.

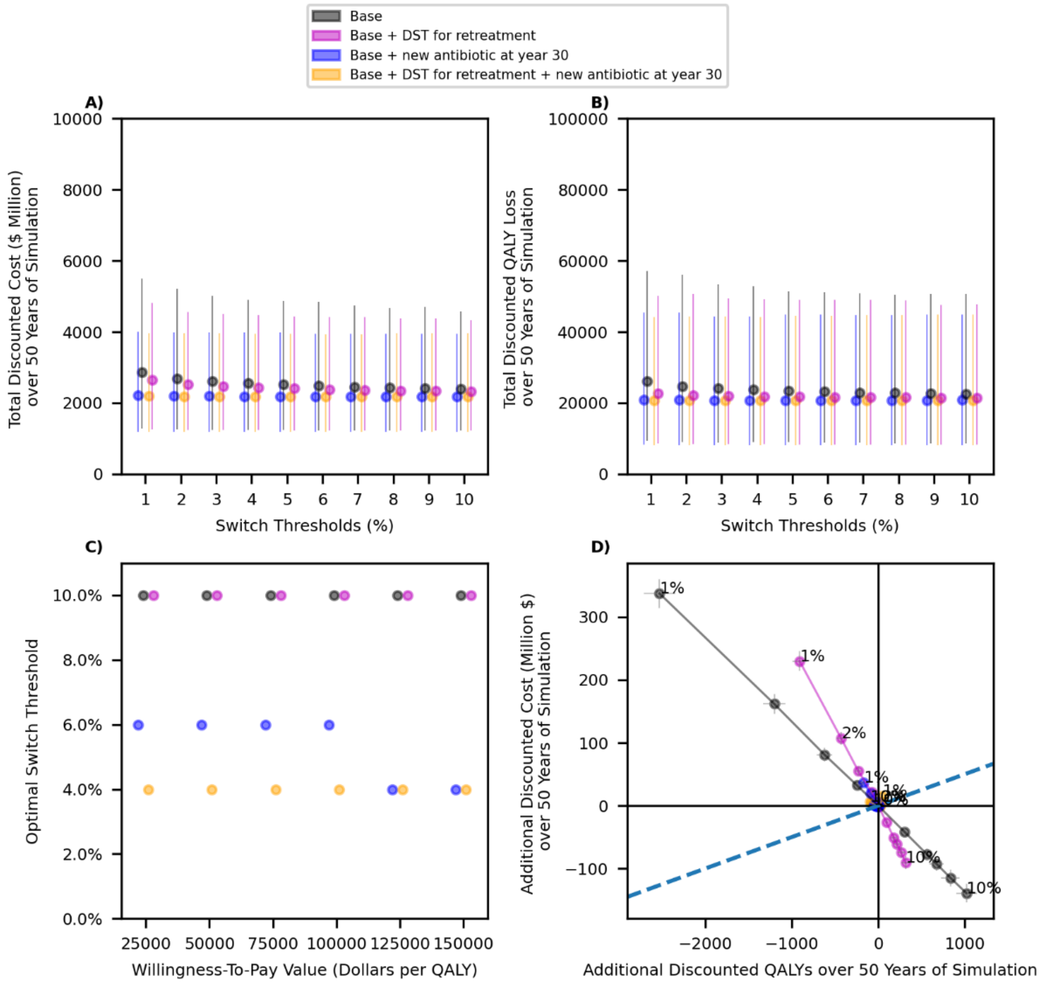

Fig D: The impact of changing the switch threshold on the total discounted cost and QALY loss over 50 years of simulation (Panels A-B), and identifying the optimal switch threshold (Panels C-D), when the cost of Drug B is twice the cost Drug A. See the caption of Fig 4 for additional information.

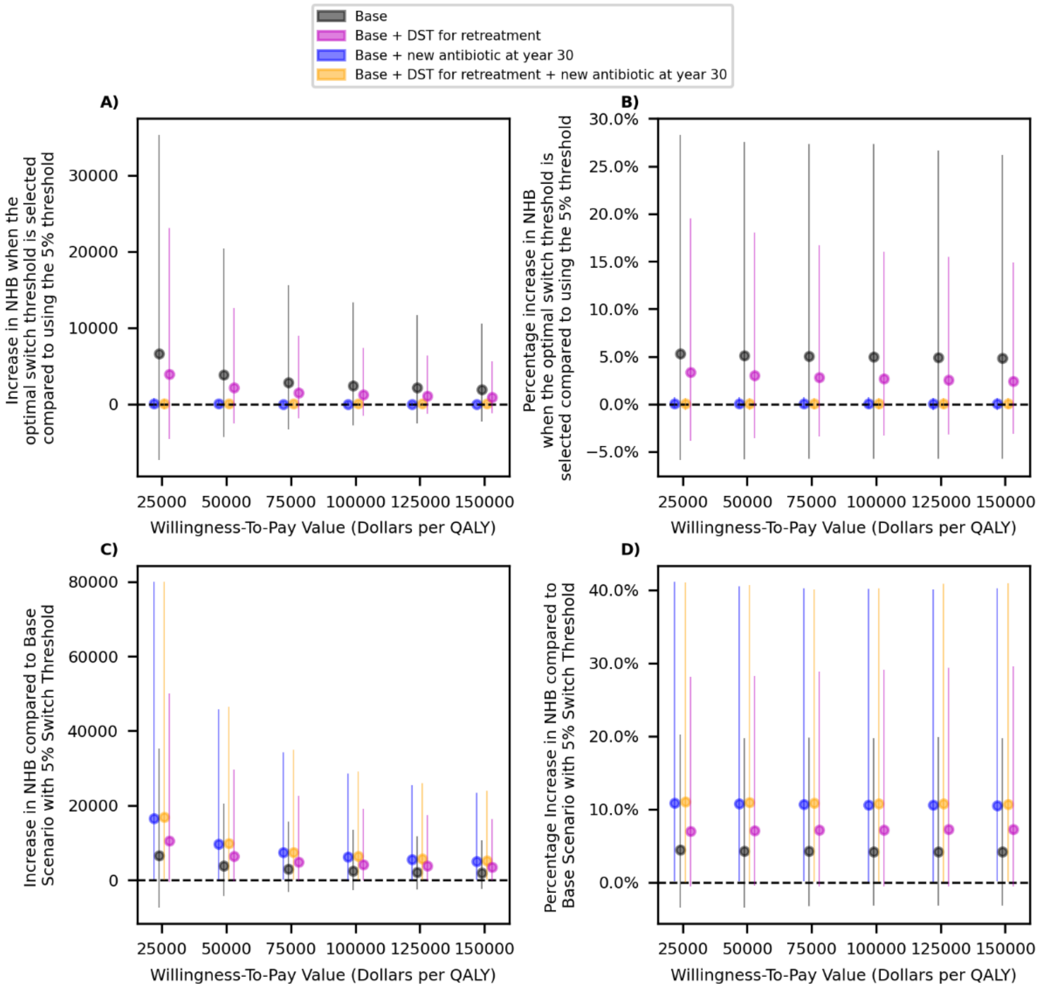

Fig E: The impact of optimizing the switch threshold (Panels A-B) and the scenarios for the availability of new antibiotics in the future (Panels C-D) on the population net health benefit (NHB) when the cost of Drug B is twice the cost of Drug A. See the caption of Fig 5 for additional information.

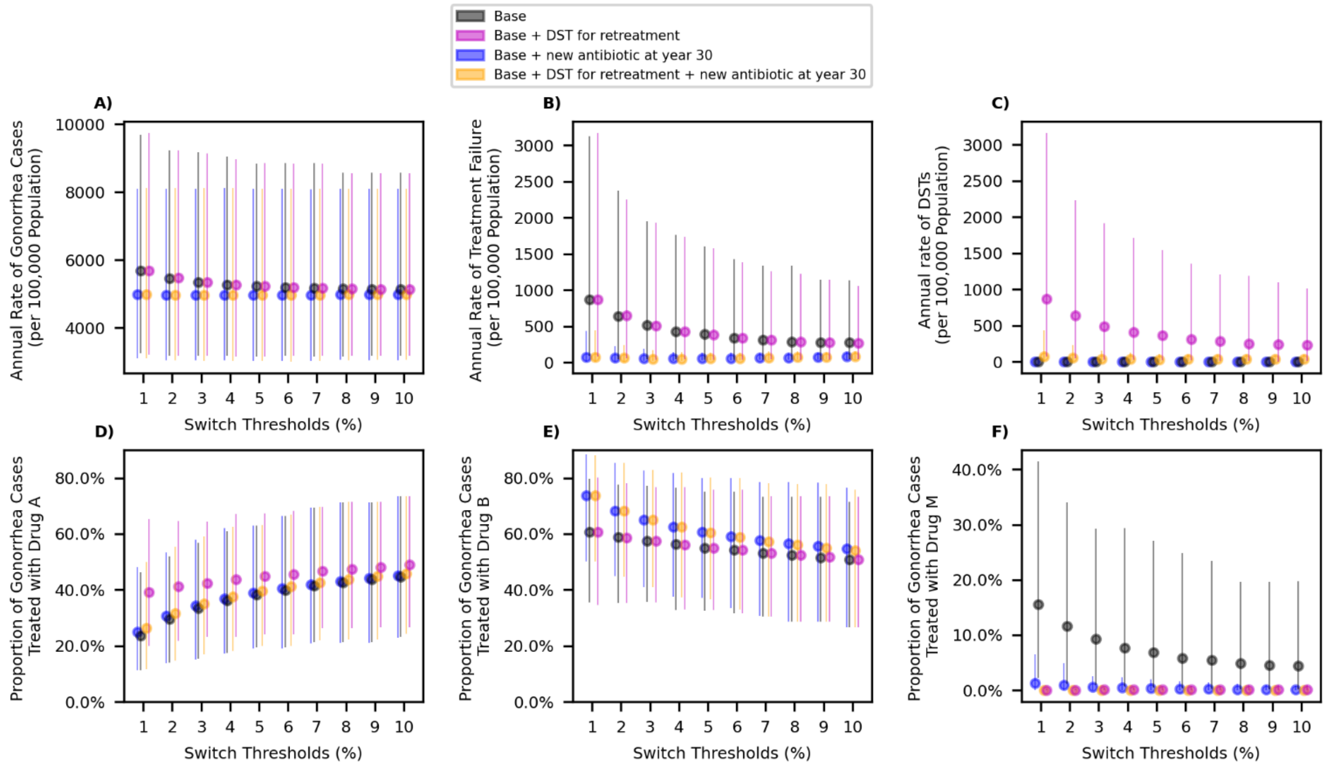

Fig F: The impact of changing the switch threshold on the average annual rate of gonorrhea cases (Panel A), average annual rate of treatment failure (Panel B), the average annual rate of drug susceptibility testing (DST) (Panel C), and the proportion of cases treated with Drug A, Drug B, and Drug M (Panels D-F), when <u>the probability that resistance develops during treatment is increased to $10^{[-5,-3]}$</u>. See the caption of Fig 3 for additional information.

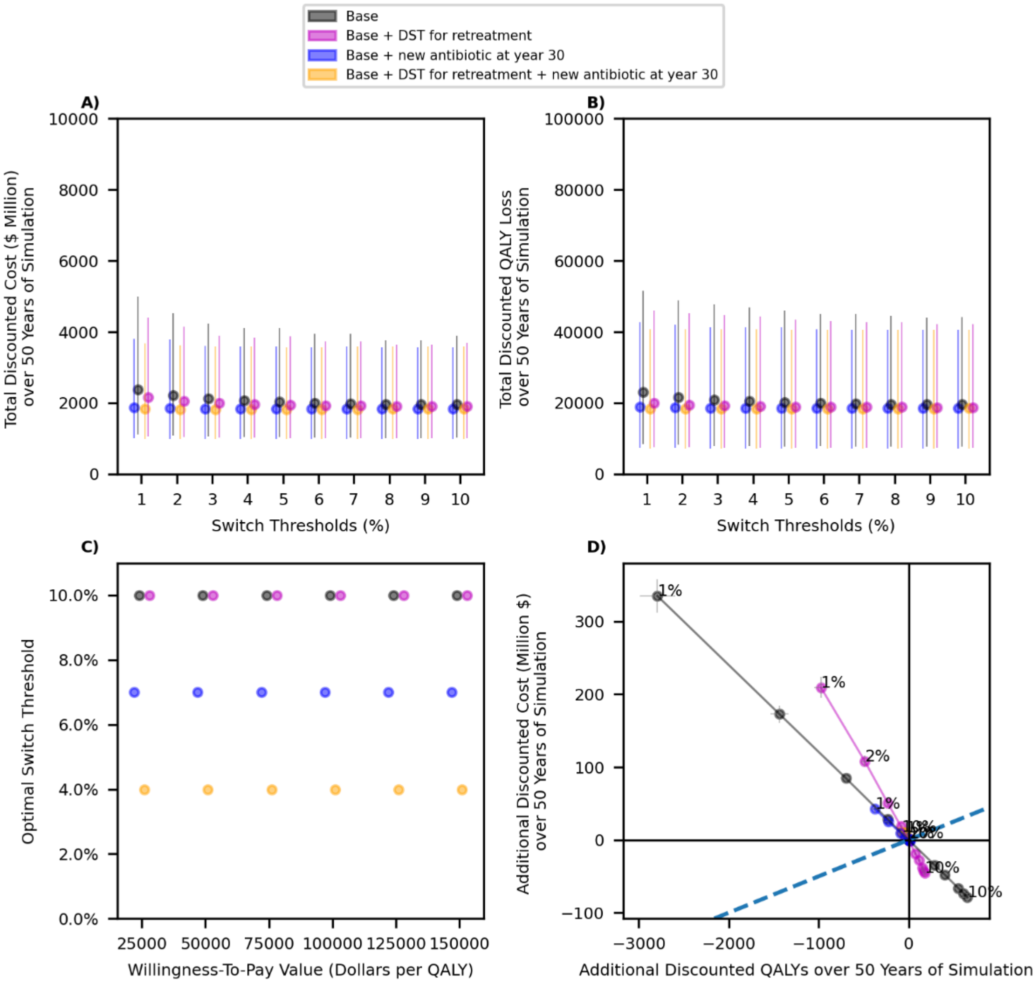

Fig G: The impact of changing the switch threshold on the total discounted cost and QALY loss over 50 years of simulation (Panels A-B), and identifying the optimal switch threshold for each scenario (Panels C-D) when the probability that resistance develops during treatment is increased to $10^{[-5,-3]}$. See the caption of Fig 4 for additional information.

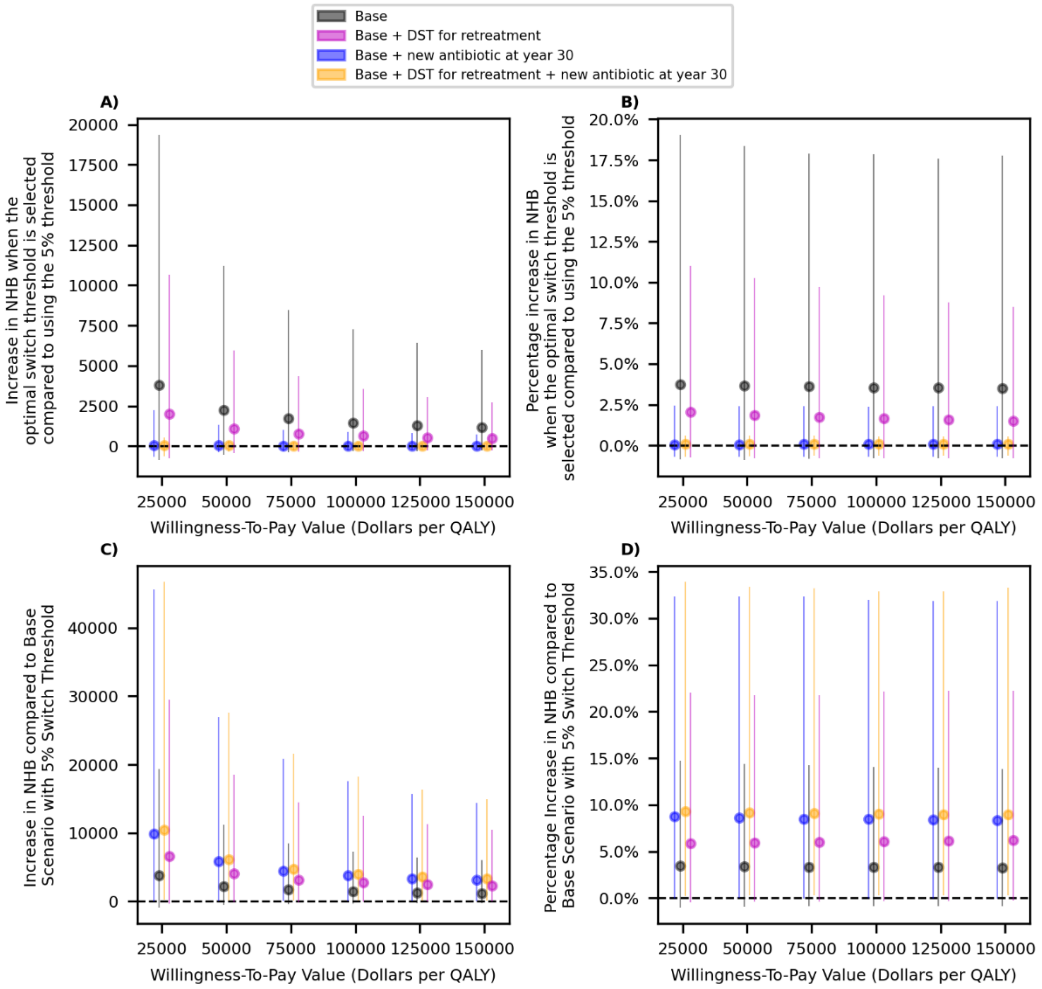

Fig H: The impact of optimizing the switch threshold (Panels A-B) and the scenarios for the availability of new antibiotics in the future (Panels C-D) on the population net health benefit (NHB) when the probability that resistance develops during treatment is increased to $10^{[-5,-3]}$. See the caption of Fig 5 for additional information.

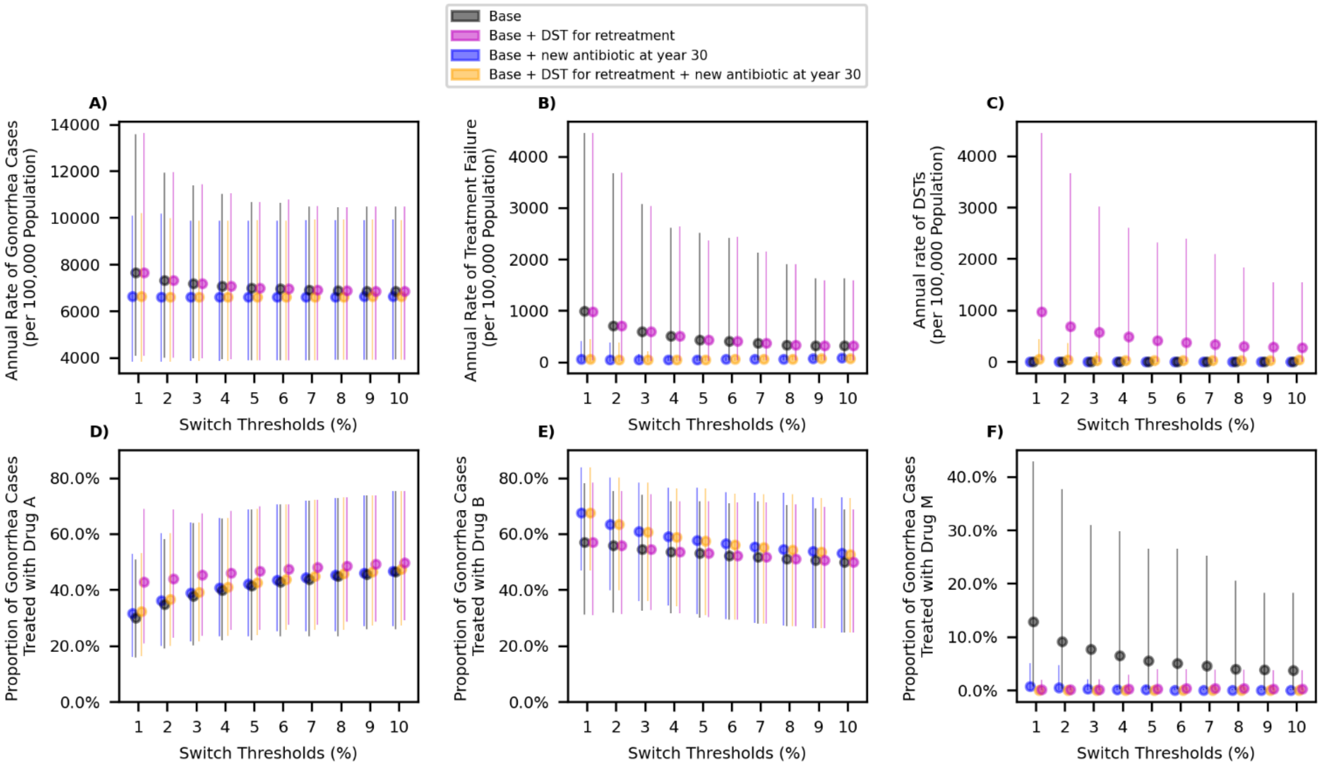

Fig I: The impact of changing the switch threshold on the average annual rate of gonorrhea cases (Panel A), average annual rate of treatment failure (Panel B), the average annual rate of drug susceptibility testing (DST) (Panel C), and the proportion of cases treated with Drug A, Drug B, and Drug M (Panels D-F) when <u>the relative transmissibility of resistant strains is [0.6, 1]</u>. See the caption of Fig 3 for additional information.

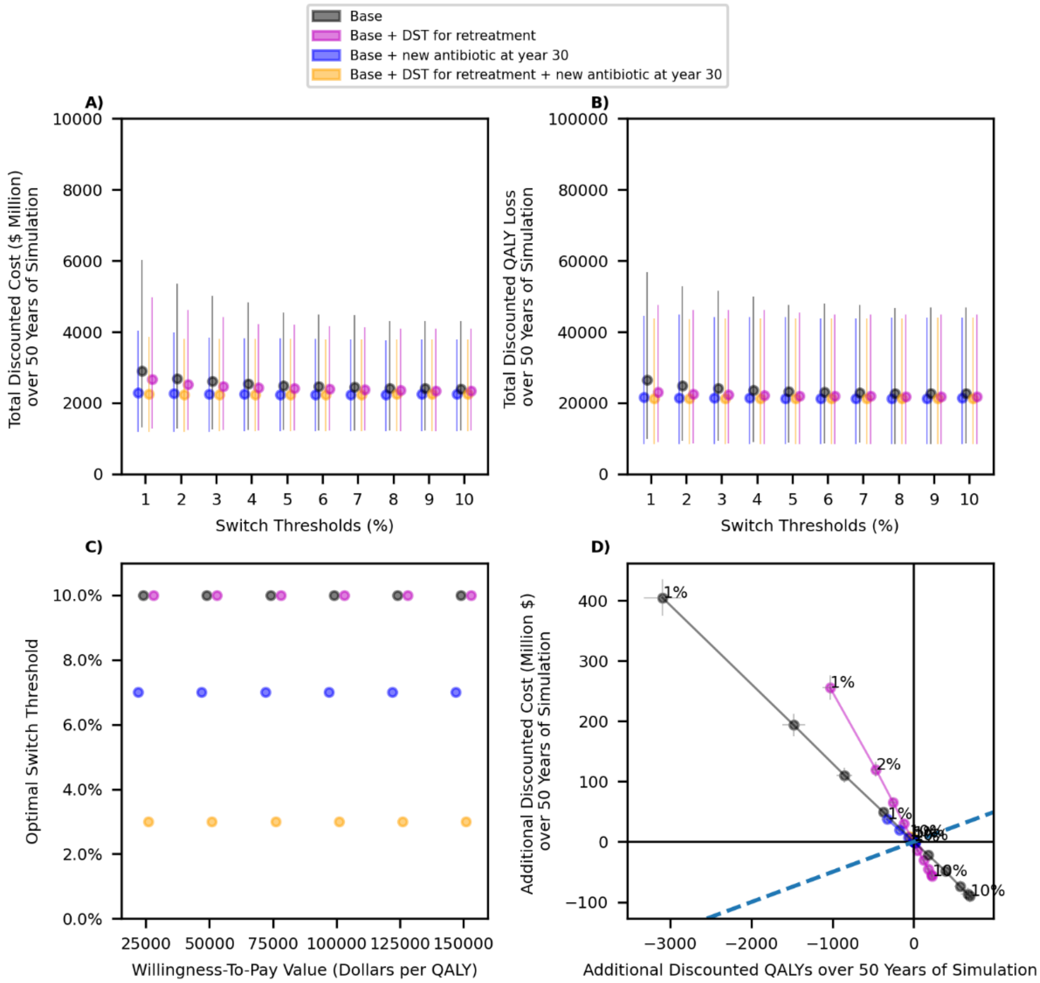

Fig J: The impact of changing the switch threshold on the total discounted cost and QALY loss over 50 years of simulation (Panels A-B), and identifying the optimal switch threshold for each scenario (Panels C-D) when <u>the relative transmissibility of resistant strains is [0.6, 1]</u>. See the caption of Fig 4 for additional information.

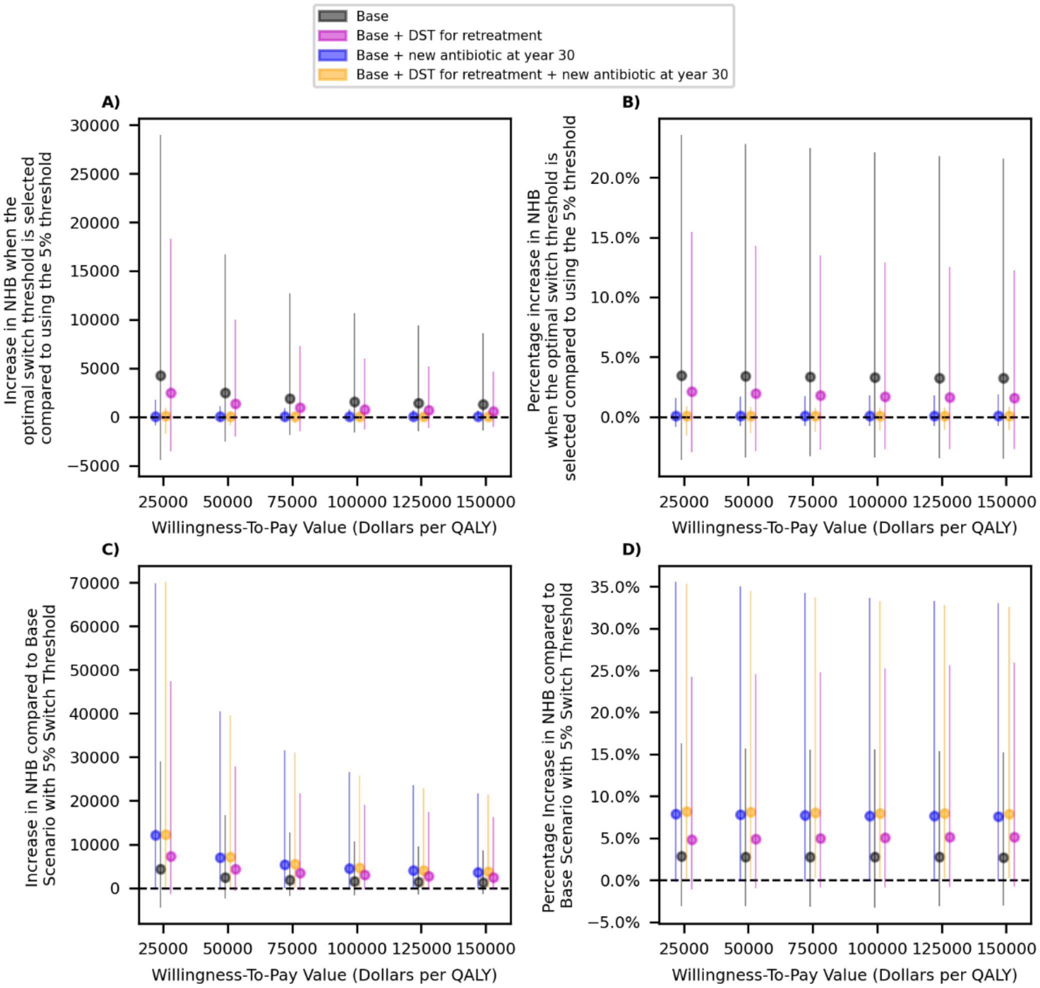

Fig K: The impact of optimizing the switch threshold (Panels A-B) and the scenarios for the availability of new antibiotics in the future (Panels C-D) on the population net health benefit (NHB) when <u>the relative transmissibility of resistant strains is [0.6, 1]</u>. See the caption of Fig 5 for additional information.

References

1. Yaesoubi R, Cohen T, Hsu K, Gift TL, Chesson H, Salomon JA, et al. Adaptive guidelines for the treatment of gonorrhea to increase the effective life span of antibiotics among men who have sex with men in the United States: A mathematical modeling study. PLoS medicine. 2020;17(4):e1003077.
2. Garnett GP, Anderson RM. Balancing sexual partnership in an age and activity stratified model of HIV transmission in heterosexual populations. Mathematical Medicine and Biology: A Journal of the IMA. 1994;11(3):161-92.
3. Jones MLJ, Chapin-Bardales J, Bizune D, Papp JR, Phillips C, Kirkcaldy RD, et al. Extragenital chlamydia and gonorrhea among community venue–attending men who have sex with men—five cities, United States, 2017. Morbidity and Mortality Weekly Report. 2019;68(14):321.
4. Centers for Disease Control and Prevention. Sexually Transmitted Disease Surveillance 2018 2018 [cited 2022 August 9]. Available from: https://www.cdc.gov/std/stats18/STDSurveillance2018-full-report.pdf.
5. Newman LM, Dowell D, Bernstein K, Donnelly J, Martins S, Stenger M, et al. A tale of two gonorrhea epidemics: results from the STD surveillance network. Public Health Reports. 2012;127(3):282-92.
6. Whittles LK, White PJ, Didelot X. Estimating the fitness cost and benefit of cefixime resistance in Neisseria gonorrhoeae to inform prescription policy: a modelling study. PLoS medicine. 2017;14(10):e1002416.
7. Tuite AR, Gift TL, Chesson HW, Hsu K, Salomon JA, Grad YH. Impact of rapid susceptibility testing and antibiotic selection strategy on the emergence and spread of antibiotic resistance in gonorrhea. The Journal of infectious diseases. 2017;216(9):1141-9.
8. Hui BB, Whiley DM, Donovan B, Law MG, Regan DG. Identifying factors that lead to the persistence of imported gonorrhoeae strains: a modelling study. Sexually transmitted infections. 2017;93(3):221-5.
9. Garnett GP, Mertz KJ, Finelli L, Levine WC, St Louis ME. The transmission dynamics of gonorrhoea: modelling the reported behaviour of infected patients from Newark, New Jersey. Philosophical Transactions of the Royal Society of London Series B: Biological Sciences. 1999;354(1384):787-97.
10. Cha S, Xia M, Finlayson T, lainn Sionean C, Teplinskaya A, Morris E, et al. HIV Infection risk, prevention, and testing behaviors among men who have sex with men National HIV behavioral surveillance 23 US cities, 2017. 2019.
